# Decay-Initiating Endoribonucleolytic Cleavage by RNase Y Is Kept under Tight Control via Sequence Preference and Sub-cellular Localisation

**DOI:** 10.1371/journal.pgen.1005577

**Published:** 2015-10-16

**Authors:** Vanessa Khemici, Julien Prados, Patrick Linder, Peter Redder

**Affiliations:** Department of Microbiology and Molecular Medicine, Faculty of Medicine, University of Geneva, Switzerland; The University of Texas Health Science Center at Houston, UNITED STATES

## Abstract

Bacteria depend on efficient RNA turnover, both during homeostasis and when rapidly altering gene expression in response to changes. Nevertheless, remarkably few details are known about the rate-limiting steps in targeting and decay of RNA. The membrane-anchored endoribonuclease RNase Y is a virulence factor in Gram-positive pathogens. We have obtained a global picture of *Staphylococcus aureus* RNase Y sequence specificity using RNA-seq and the novel transcriptome-wide EMOTE method. Ninety-nine endoribonucleolytic sites produced *in vivo* were precisely mapped, notably inside six out of seven genes whose half-lives increase the most in an RNase Y deletion mutant, and additionally in three separate transcripts encoding degradation ribonucleases, including RNase Y itself, suggesting a regulatory network. We show that RNase Y is required to initiate the major degradation pathway of about a hundred transcripts that are inaccessible to other ribonucleases, but is prevented from promiscuous activity by membrane confinement and sequence preference for guanosines.

## Author Summary

Ribonucleic acids (RNA) are key intermediates between the heritable genome and the expression of proteins. Thus the level of a specific RNA determines the capability of the cell to produce the corresponding protein. Bacteria maintain a very high turnover (high production but also high decay-rate) of RNA molecules, to ensure that they can, at any time, change the protein production program in response to outside stimuli. However, all types of RNA molecules do not have the same half-life, some last for tens of minutes whereas most only last for seconds. Here we examine how endoribonuclease Y (RNase Y), one of the enzymes that cleave RNA into smaller fragments, chooses its targets from the pool of cellular RNA. We show that RNase Y prefers to cleave RNA at a specific ribonucleotide sequence, thus keeping the RNA degradation to the correct level, not too fast and not too slow. RNase Y is anchored to the inside of the cell envelope, and we furthermore show that releasing RNase Y into the interior of the cell results in poor growth, but is at the same time able to compensate for the loss of CshA, another factor in the RNA decay machinery.

## Introduction

RNA synthesis and decay are both vital factors in the bacterial response to changing growth conditions, by starting up and shutting down cellular programs, respectively. RNA synthesis is determined by promoter strength, via the adherence of the RNA polymerase sigma factor to promoter motifs such as the -10 and -35 elements, and can be further regulated by transcriptional regulators. RNA decay is a highly efficient process in bacteria, and most mRNAs have half-lives of less than a few minutes [1], which allows the cell to rapidly shut down unwanted protein production. In *Escherichia coli*, the rate-limiting step in the RNA degradation process of the majority of RNA is thought to be an endoribonucleolytic cleavage performed by RNase E, after which, a collection of exonucleases can convert the resulting RNA fragments to single nucleotides [2]. Surprisingly for such a fundamental function, RNase E initiated decay is not ubiquitous in bacteria, since RNase E homologs are absent from many Firmicutes, including pathogens like Staphylococci and Streptococci, as well as ε-Proteobacteria and Spirochaetales [3]. Instead it has recently been proposed that in Firmicutes, the initiating cleavage is performed by the endoribonuclease RNase Y [4–6].

RNase Y, encoded by the *cvfA* (or *rny*) gene, was originally discovered in *Staphylococcus aureus* and *Streptococcus pyogenes* as an important virulence factor [7]. More recently, *S*. *aureus* RNase Y was shown to be responsible for maturation of the *sae* virulence regulating operon [8] and in *Bacillus subtilis* to cleave the SAM-riboswitches, and mature RNase P RNA and scRNA [4,9]. An N-terminal hydrophobic alpha-helix anchors RNase Y to the membrane [10], and a coiled-coil domain then separates the anchor from an RNA binding KH domain and a HD domain containing the Histidine-Aspartate active site [4,7,11]. RNase Y forms dimers and even tetramers via the coiled-coil domain and the membrane anchor, independently of each other [11].

In contrast to RNase E, the RNase Y gene is not essential, and can be deleted in *S*. *aureus*, *S*. *pyogenes* and *B*. *subtilis* [7,8,12–14]. The fitness cost of these RNase Y deletions range from mild to none in *S*. *aureus* and *S*. *pyogenes*, but is quite severe in *B*. *subtilis*, to a point where the gene was originally thought to be essential [4,6,11]. However, the pathogenesis of both *S*. *pyogenes* and *S*. *aureus* is severely affected by the deletion of RNase Y, in both silkworm and mouse models [7,8,14], cementing the importance of RNase Y across a wide range of Firmicutes.

Several functions, that are not mutually exclusive, have been described for the *S*. *aureus* RNase Y: A 3' phosphodiesterase activity, demonstrated *in vitro* [15,16], utilises the same active site in the HD domain as the endoribonucleolytic activity which has been demonstrated *in vivo* by the identification of a single RNase Y cleavage site in the *sae* virulence regulator operon [8]. *S*. *aureus* RNase Y has furthermore been shown by bacterial-two-hybrid assay to interact with enolase, a key metabolic enzyme, and the DEAD-box RNA helicase CshA [17], which is thought to open secondary RNA-structures in an ATP-dependent manner [18]. Both interaction has recently been confirmed *in vivo*, by tandem affinity purification, to be via the non-enzymatic C-terminal extension of CshA [19]. CshA has been proposed to help RNases in the RNA decay machinery gain access to cleavage sites that are buried within secondary structures [17,20]. When the *cshA* gene is deleted in *S*. *aureus*, the mutants grow very poorly at low temperature (24°C) and the decay of about a hundred RNAs is retarded, including the quorum sensing *agr* mRNA which indirectly promotes hemolysis [18,19]. In this respect, RNase Y seems to be functionally similar to RNase E that serves as a scaffold for the *E*. *coli* degradosome [21].

A number of additional RNases are involved in general RNA decay in *S*. *aureus* (as opposed to maturation of stable RNAs), supported either by experimental evidence or inference by homology. There are three identified 3' to 5' exoribonucleases, polynucleotide phosphorylase (PNPase), RNase R, and SA1660/YhaM [22]. RNase R is essential in *S*. *aureus* but has not been studied [23], whereas a transcriptome-wide decay assay has been made with a PNPase mutant of *S*. *aureus*, showing that many RNAs are stabilised [1]. The importance of PNPase and RNase R is also evident in *E*. *coli*, where the single knockout mutants are viable whereas the double knockout is lethal [24], indicating redundancy in the 3' exoribonucleolytic pathway. In contrast to *E*. *coli*, a large number of bacterial phyli, including the Firmicutes, encode one or two RNase J homologs. These are 5' to 3' exoribonucleases, an activity that until recently was thought to be absent from bacteria. *S*. *aureus* encode two paralogs, RNase J1 and RNase J2, that form a complex where only the active site of RNase J1 is responsible for the 5' exoribonucleolytic activity *in vivo* [17,25]. Deletion mutants of either RNase J exhibits very severe growth phenotypes and the correct maturation of essential molecules such as 16S rRNA and RNase P RNA are dependent on the RNase J1/J2 complex [25], however these RNases have not been studied carefully in terms of RNA decay.

We here examine the role of RNase Y in *S*. *aureus* by transcriptome-wide RNA decay analyses, identifying increased RNA half-lives that are due to RNase Y dependent degradation defects. We additionally show that the N-terminal membrane anchor is not needed for the RNase Y endoribonucleolytic activity *in vivo*, and that its function must therefore be to sequester either RNase Y itself, or a protein partner(s), to the membrane.

Finally, even in the well-studied *E*. *coli* system, where RNase E has been studied for decades, it remains unclear how certain RNA substrates are chosen for degradation more readily than others, since multiple signals, at both the 5'-end and at the RNase E cleavage site, appears to be of importance [2,3]. Even less is known about RNase Y, and we therefore use the novel global method EMOTE (Exact Mapping Of Transcriptome Ends) [25] to identify, to our knowledge for the first time, the *in vivo* recognition sequence motif of a decay-initiating endoribonuclease, and are able to show that the preferred cleavage site is 3' of a guanosine, in an otherwise adenosine and uridine rich region.

## Results

### RNA decay of a specific sub-set of the transcriptome is affected by deletion of RNase Y

In order to understand the phenotypes observed in *S*. *aureus* RNase Y mutants, two previous transcriptome-wide studies have observed altered steady state levels of a number of transcripts [8,16]. These effects were consistent with a defect in RNA degradation, and the half-lives were examined for a small number of transcripts, some of which did indeed show increased RNA stability [8]. However, the global scale of half-life increase–and thus the scale of RNA decay rate-limiting activity performed by RNase Y–cannot be discerned by steady state level measurements alone, since indirect effects of the RNase Y deletion may cause an increase or decrease in promoter activity. We therefore performed a transcriptome-wide RNA degradation assay to understand whether the increased steady-state RNA levels in a strain deleted for the RNase Y gene (ΔY) are indeed caused by an increase in RNA half-life. Decay of transcripts in the ΔY strain was measured by treating exponentially growing cultures with rifampicin to inhibit the RNA polymerase, whereupon samples were taken at 0, 150, 300 and 600 seconds. The isolated RNA for each time point was sequenced using the stranded Illumina RNA-seq protocol. Using the normalisation and criteria described in the Experimental Procedures we were able to obtain the half-life of RNA corresponding to 1464 ORFs, and the 0 second time-point was additionally used to measure the steady-state levels. The assay was carried out in duplicates and compared to isogenous wild-type (WT) data obtained previously [19]. Differences in RNA levels and stability between WT and ΔY were determined using a smallest-difference model: For each ORF, the calculated steady state levels and half-life for the two WT and the two ΔY datasets were compared, and the smallest difference between WT and ΔY was used. If the range of WT and the range of ΔY overlapped, then the difference was set to zero. The smallest-difference model is well suited to experiments with a low number of biological replicates, and gives a conservative estimate of the number of affected genes.

**Fig 1:**
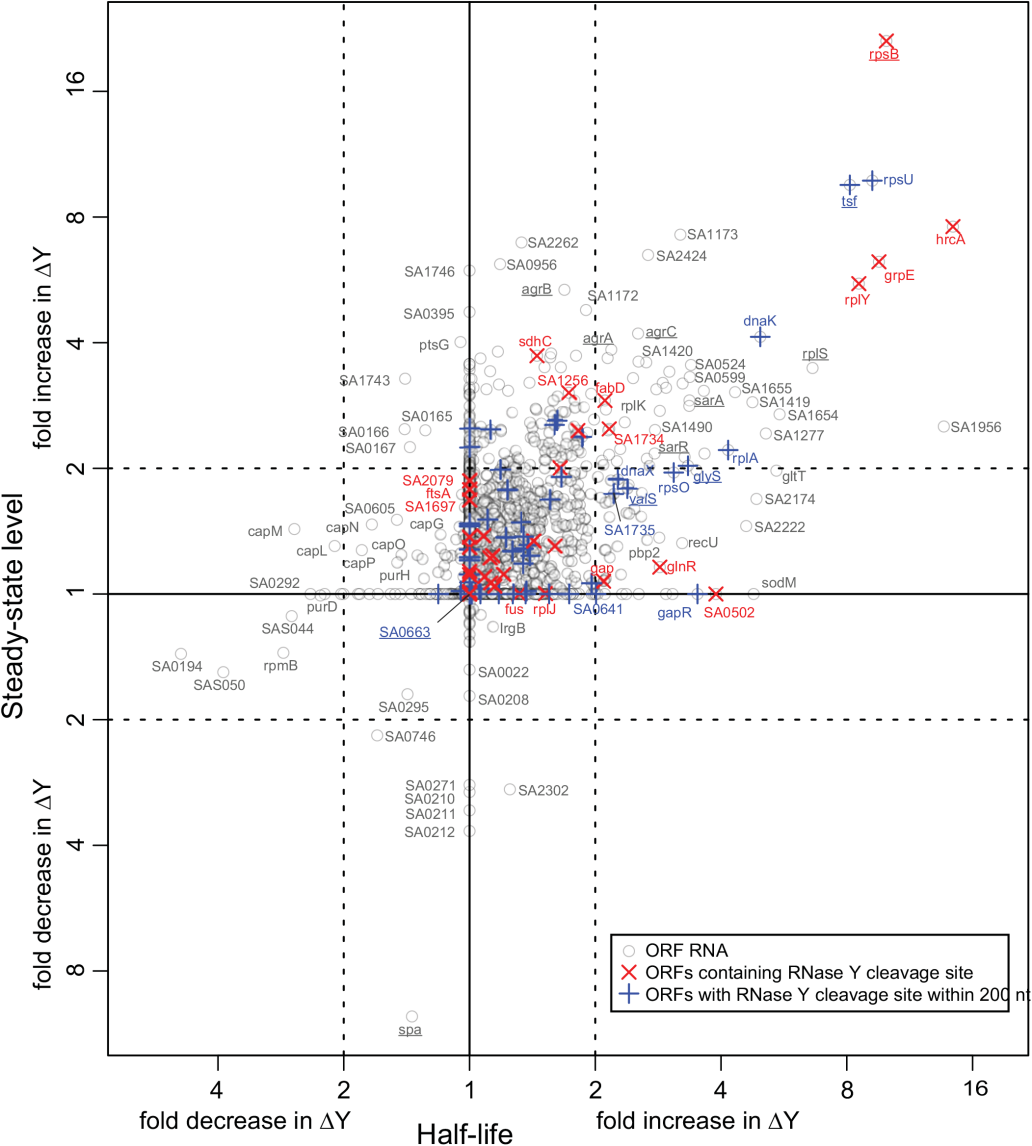
Correlation between changes in half-lives and steady-state levels. The change between WT and ΔY in the half-life of each ORF was estimated using a smallest-difference model, and the same was done for the steady-state levels of the ORFs. The fold-change in half-life is plotted on the x-axis and the fold-change in steady-state level on the y-axis. Each grey circle represents an ORF transcript where sufficient data was available (1464 in all), and the names of ORFs discussed in this study are underlined. Red X's show which ORFs contain an identified RNase Y cleavage, and blue +'s show the ORFs where an RNase Y site was identified within 200 nt of an ORF.

Only 248 ORF RNAs are strongly affected by the RNase Y deletion, whereas 160 ORFs increase in stability just below the conservative 2 fold cut-off used throughout this study (summarised in Table 1, see also S1 Table for a full list). There was an overall good correlation between increased RNA stability and increase in steady-state levels as shown in Fig 1. The ORFs that were significantly affected in both steady-state and half-life levels (upper right quadrant in Fig 1) comprise 50 ORFs encoding several ribosomal proteins, housekeeping genes, chaperones, as well as the virulence regulators *sarA*, *sarR*, *agrA* and *agrC*. The correlation is nevertheless not absolute and some ORFs that accumulate are not stabilised, presumably due to indirect effects of the ΔY mutation. There are even seven ORF RNAs that decrease in abundance, notably the >8 fold lower expression of the *spa* transcript, which encodes the antibody-binding Protein A (Fig 1). However *spa* is negatively regulated by the *agr* quorum-sensing system [26] whose transcript is stabilised and accumulate in the ΔY mutant (see above), providing a logical explanation and a perfect example of an indirect effect.

**Table 1:** Overview of the steady-state level and stability data.

		Stability fold-change					
	Number of ORF RNAs:	>2 down	>1.5 and <2 down^a^	Unchanged	>1.5 and <2 up^a^	>2 up	Total
Steady- state level fold- change	>2 down	0	1	6	0	0	7
	>1.5 and <2 down^a^	1	0	3	0	0	4
	Unchanged	10	8	877	74	25	994
	>1.5 and <2 up^a^	0	0	215	39	19	273
	>2 up	0	0	89	47	50	186
	Total	11	9	1190	160	94	1464^b^
		Stability fold-change					
	Number of ncRNAs:	>2 down	>1.5 and <2 down^a^	Unchanged	>1.5 and <2 up^a^	>2 up	Total
Steady- state level fold- change	>2 down	0	1	1	0	0	2
	>1.5 and <2 down^a^	0	0	0	0	0	0
	Unchanged	2	2	29	4	1	38
	>1.5 and <2 up^a^	0	1	6	2	1	10
	>2 up	0	0	7	4	6	17
	Total	2	4	43	10	8	67^b^

^a^) Changes below 2 fold are not considered significant for this study.

^b^) Only RNAs that passed the quality criteria are included.

Non-coding RNAs are important components of bacterial gene regulation. The list of non-coding (ncRNAs), small RNAs and untranslated regions (UTRs) compiled by Sassi and coworkers [27] (collectively referred to as ncRNAs throughout this manuscript, for simplicity) were therefore added to the analysis of open reading frames described above (S1 Fig, S1 Table). Only 67 ncRNAs passed the quality control criteria for confidently estimating their half-lives, mainly due to the silica-column based method of RNA isolation used for the RNA-seq, which depletes RNA molecules shorter than 200 nucleotides (nt). Among them, six ncRNAs (srn_1510_rsaA, srn_1520_Sau76, srn_2620_Teg61, srn_2830_SbrB, srn_3820_SprX and srn_3160_RsaOD/TB-glyS) were stabilised and accumulated above the 2-fold cut-off (S1 Table). As expected, abundance of the 514 nt regulatory RNAIII was increased in ΔY, in concordance with the increase in *agr*, which drives the RNAIIII promoter, and the decrease in *spa*, whose decay is initiated by RNAIII [28].

### Identifying RNase Y cleavage sites by EMOTE

The identification of RNA molecules that are stabilised and/or accumulated in ΔY, naturally leads to the question of whether these RNAs are cleaved directly by RNase Y, and if so, where on the RNA the cleavage takes place. Knowing the exact endonucleolytic sites might reveal which type of signals regulates RNase Y activity. However, until now, only a few RNase Y cleavage sites have been mapped in *B*. *subtilis*, and only one single site has been located in *S*. *aureus* [3,8,9]. Therefore we used Exact Mapping Of Transcriptome Ends (EMOTE), a technique that maps the exact 5'-end of mono-phosphorylated RNA on a transcriptome-wide scale [29], to identify RNase Y cleavage sites in *S*. *aureus*.

Briefly the EMOTE assay consists of ligating a synthetic RNA oligo (Rp6) to the 5'-ends of total RNA, using T4 RNA ligase 1, which will only accept 5' mono-phosphorylated RNAs as substrate. The ligated RNA is then reverse transcribed to cDNA, and by using the sequence of Rp6 as anchor for Illumina sequencing, it is possible to specifically sequence only the 5'-ends of mono-phosphorylated cellular RNA. The large number of reads generated by Illumina sequencing can then be mapped onto the genome of the organism, and the exact positions of each of the mono-phosphorylated 5'-ends are obtained (see S2 Fig and Materials and Methods).

Mutants where the entire RNase Y gene (*cvfA*) of *S*. *aureus* is deleted have previously been generated by our group and others [7,8,12]. Studies have indicated that RNase Y is part of a network of protein-protein interactions involved in RNA decay [17], and it is therefore possible that deleting the entire gene causes secondary effects. We therefore replaced the wild-type RNase Y allele with a mutated gene in which the active site HD-motif (Histidine 367 and Aspartate 368) was replaced by two Alanines, resulting in strain Y^367AA^. Additionally, the putative N-terminal membrane anchor in RNase Y (amino acids 2–24) was also deleted by allelic replacement, while preserving the alternative GTG start-codon of the RNase Y gene, generating strain Y^Δ2–24^. Growth of both the RNase Y deletion strain (ΔY) and Y^367AA^ were very similar to the WT strain, whereas strain Y^Δ2–24^, with an anchorless RNase Y, grew markedly slower (S3 Fig).

EMOTE was performed in duplicate with WT and the different RNase Y mutants, whereupon comparison of EMOTE assays from WT with the combined data from ΔY and Y^367AA^ strains was used to map potential endoribonucleolytic cleavage sites that were RNase Y dependent, in other words, 5'-ends that were observed in the WT strain, but not in the ΔY and Y^367AA^ mutants.

The highly stringent conditions used (see Materials and Methods) resulted in a list of 99 RNase Y cleavage sites, of which 50 are inside annotated open reading frames and 49 are either in UTRs, ncRNAs or intergenic regions (S2 Table). Conservative criteria were chosen in order to avoid false positives, however even if more lenient criteria were used, such a list would not (and cannot be) exhaustive, due to a number of factors that are unavoidable in a transcriptome-wide *in vivo* assay. a) Cleavage sites in transcripts with low abundance will be filtered out, since the signal in the WT will be relatively weaker. b) It is possible, and even likely, that RNases overlap in their activity *in vivo*, similar to what has been observed for *B*. *subtilis* RNase Y and RNase J1 *in vitro* [3], therefore any sites that are also cleaved by alternative RNases will be filtered out. c) A potential site will only be detected if the downstream fragment is stable enough to remain detectable in the cell, a problem that is increased in organisms such as *S*. *aureus*, which have RNase J with 5' to 3' exoribonuclease activity that can remove the 5'-end generated by RNase Y as soon as it is made. This problem could possibly be avoided by generating a ΔYΔJ1 mutant and use a ΔJ1 mutant for comparison, but unfortunately we have after several attempts been unsuccessful in producing such a double mutant. Furthermore, it cannot excluded that the 5'-ends interpreted as RNase Y cleavage sites are in fact caused by another RNase whose activity is altered in the RNase Y mutants. This could be an undiscovered endoribonuclease which is inactive in the absence of RNase Y activity, or possibly that the exonucleolytic activity of RNase J stalls at certain positions in the WT strain, whereas no such stalling occurs in the ΔY strains. To exclude the latter possibility, the EMOTE data for a ΔJ1ΔJ2 mutant were examined. In this strain there are no RNase Js [25], which can consequently not stall, and the presence of EMOTE reads mapping to 97 of the 99 RNase Y cleavage positions proves that the vast majority of the identified RNase Y sites are not caused by variations in RNase J stalling.

**Fig 2:**
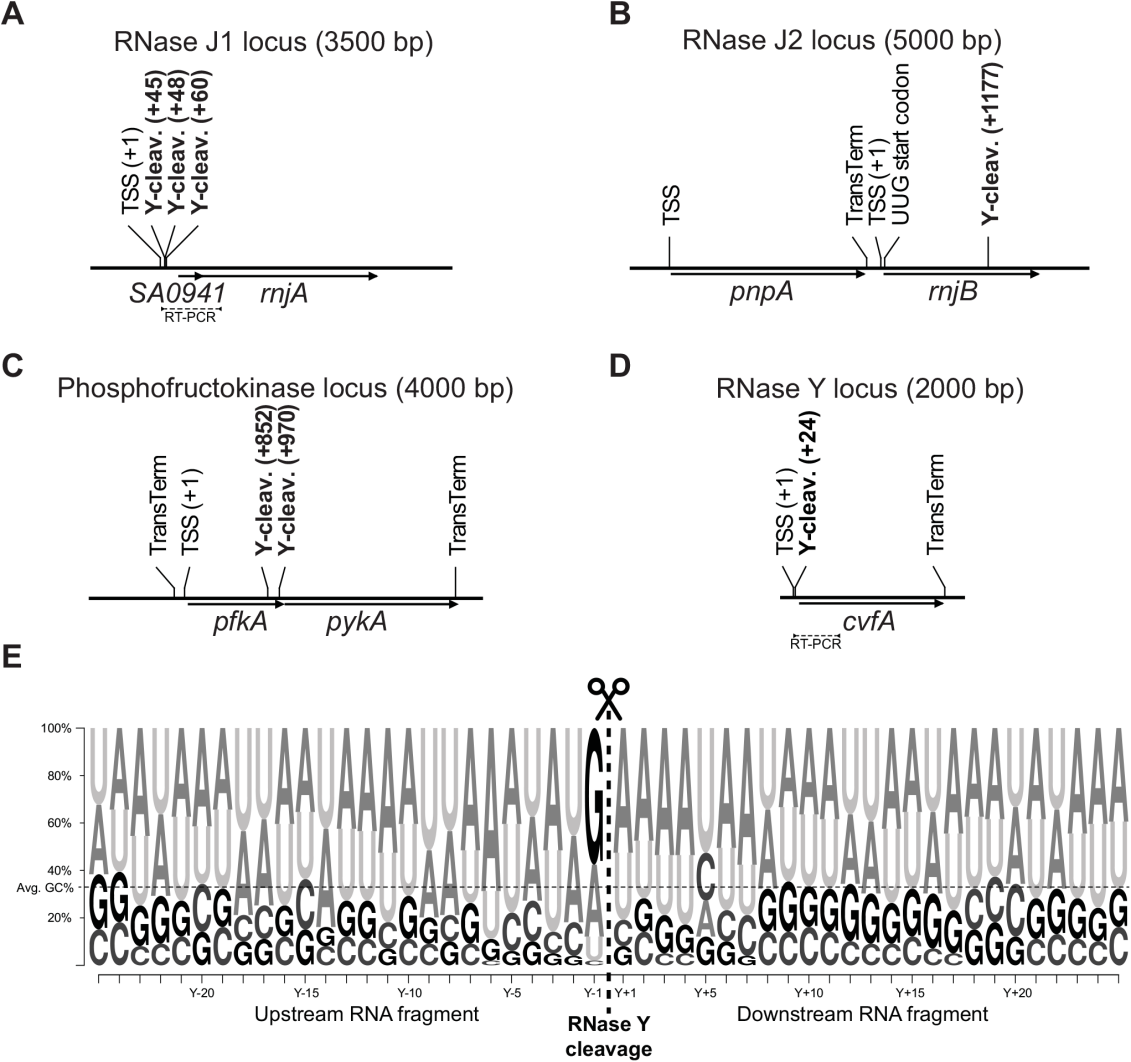
RNase Y cleavage sites fall within or near the genes encoding components of the putative degradosome, as defined by [17], and show sequence preference for guanosines. Predicted transcription start sites (TSS), detected RNase Y cleavage sites (Y-cleav.) and predicted transcriptional terminators (TransTerm) are indicated. Thin dotted lines show the sequence amplified by RT-PCR to demonstrate that RNase Y cleavage sites and ORFs are potentially on the same RNA molecules. (A) *SA0941-rnjA* operon, encoding a hypothetical protein and RNase J1. (B) *rnjB* gene encoding RNase J2 (*pnpA* encodes PNPase). (C) *pfkA-pykA* operon, encoding phosphofructokinase and pyruvate kinase. (D) The RNase Y gene (*cvfA*) with the putative RNase Y cleavage site at position +24. (E) RNase Y cleaves preferentially after a purine. 25 nucleotides on each side of the 99 RNase Y sites (see S2 Table) were extracted from the *S*. *aureus* N315 genome, and a frequency plot was generated. The RNase Y cleavage site (thick vertical dotted line), and the position detected by EMOTE (Y+1) are indicated. The preference for a guanosine residue immediately prior to the RNase Y cleavage sites is evident. Additionally, there is an enrichment of A's at position Y–6 and of pyrimidines at position Y+5. Finally, it appears that A's or U's are favoured in the region from 11 nt upstream to 7 nt downstream of the cleavage site, however this could also be due to the over-all nucleotide composition of *S*. *aureus*, which has 33% G+C (thin horizontal dotted line).

One identified cleavage site corresponded to the previously demonstrated RNase Y cleavage in the *sae* operon [8]. A number of the identified RNase Y cleavage sites fell within nine ORFs that were stabilised in our transcriptome wide RNA stability assay, showing that these coding RNAs are direct targets of RNase Y, which performs the cleavage that initiates degradation (Table 2, Fig 1). However, a decay-initiating cleavage does not need to be within an ORF, but can in principle be anywhere on the RNA. Therefore the search was extended 200 nucleotides in either direction from the ORFs, which identified an additional ten stabilised ORFs with associated RNase Y cleavage sites. To ensure that these ORFs are potentially on the same RNA molecules as the detected RNase Y cleavage, reads were identified in the RNA-seq data that span both the cleavage site and the ORF, with >50 reads considered significant. An RT-PCR assay was used to connect those sites/ORFs that either have too few RNA-seq reads or are further from the ORF than the length of the Illumina reads (50 nt). Combined, the RT-PCR reactions and the RNA-seq connected 37 cleavage sites with corresponding ORFs (S4 Fig), including all ORFs that are significantly stabilised in the RNase Y mutant. Thus a total of 19 stabilised ORFs (20%) could be linked to one or more of the 99 identified RNase Y cleavage sites. Moreover, RNase Y cleavage sites were identified in or near RNase J1 (*SA0941-rnjA* operon), RNase J2 (*rnjB*) and phosphofructokinase (*pfkA-pykA* operon), which have all been linked to the RNA decay machinery (Fig 2A–2C) [17]. Finally, because the RNase Y-encoding *cvfA* gene is not expressed in the ΔY strain it is inherently excluded by the criteria used to identify RNase Y cleavage sites, however, when EMOTE data from the Y^367AA^ strain (which does express *cvfA*) is compared to WT data, then a potential RNase Y cleavage site inside its own transcript is revealed (Figs 2D and S4; Table 2).

**Table 2:** Stabilised and degradosome ORFs with an associated RNase Y cleavage site.

ORF	Start^a^	End^a^	Length	Strand	Number of RNase Y sites inside the ORF	Distance to the nearest RNase Y site^b^	Half-life change (fold)	Steady-state change (fold)
Stabilised ORFs with an associated RNase Y cleavage site								
*dnaX*	502594	504291	1698	+	0	22	2.3	1.9
*rplY*	529681	530334	654	+	1	0	8.5	5.5
*rplA*	576747	577439	693	+	0	12	4.2	2.2
*SA0502*	587068	587322	255	+	1	0	3.9	1
*SA0641*	735417	735860	444	-	0	-144	2.0	1
*gapR*	831619	832632	1014	+	0	1	3.5	1
*Gap*	832685	833695	1011	+	1	0	2.1	1.1
*fabD*	1213830	1214756	927	+	1	0	2.1	2.9
*rpsB*	1245199	1245966	768	+	1	0	9.9	21.1
*Tsf*	1246148	1247029	882	+	0	-106	8.1	9.5
*rpsO*	1265805	1266074	270	+	0	74	3.1	2.0
*glnR*	1306778	1307146	369	+	1	0	2.9	1.2
*glyS*	1600376	1601767	1392	+	0	-115	3.3	2.0
*rpsU*	1608721	1608897	177	-	0	-35	9.2	9.8
*dnaK*	1613514	1615346	1833	-	0	-18	5.0	4.1
*grpE*	1615415	1616041	627	-	1	0	9.6	6.2
*hrcA*	1616073	1617050	978	-	1	0	14.4	7.6
*valS*	1693943	1696573	2631	-	0	-181	2.4	1.8
*SA1734*	1986743	1987303	561	+	3	0	2.2	2.5
*SA1735*	1987356	1988285	930	+	0	-168	2.2	1.7
RNase Y cleavage sites associated with degradosome ORFs								
*SA0941*	1068429	1068599	171	-	0	-129, -141, -144	1^c^	1.5^c^
*rnjA*	1066732	1068429	1698	-	0	-347, -359, -362	1.2	1
*rnjB*	1268775	1270448	1674	+	1	0	1.3^c^	1.1^c^
*pfkA*	1736036	1736959	924	-	2	0	0	0
*cvfA*	1281967	1283526	1560	+	0	-54	ND^d^	ND^d^

a) Genome positions from *S*. *aureus* N315 (NC_002745.2) are used throughout the manuscript.

b) On the same strand and shown by RT-PCR or RNA-seq to be potentially on the same RNA molecule, 0 = Cleavage is inside the ORF, negative numbers indicate upstream cleavage.

c) Did not pass the quality criteria.

d) Not determined.

We were unable to identify any convincing RNase Y cleavage sites in the rRNA, and it is possible that neither maturation, nor decay, of the ribosomes involve a specific role for RNase Y. However, it cannot be excluded that RNase Y contributes to these processes with activity that overlaps with other RNases.

To further analyse the fate of the two fragments generated by RNase Y cleavage, a region of 300 nt upstream and a region of 300 nt downstream of each RNase Y site were defined, and their stability and steady-state levels were examined. Based on this data, all but one of the RNase Y sites could be organised into four subgroups, depending on the fate of either the upstream or the downstream fragment in the WT strain, compared to the ΔY strain (S2 Table, S5 Fig): Class 1 (17 sites), where both upstream and downstream fragments were stabilised by the lack of cleavage (i.e. in the ΔY mutant), presumably indicates that the RNase Y cleavage is an initiating event for decay of the entire RNA. Class 2, (61 sites), probably represents a maturation event, since neither the stability of the upstream, nor the stability of the downstream fragment are affected by the cleavage, however, it may still be regulatory if the cleavage site is situated inside an ORF. Class 3 and 4 (12 and 8 sites, respectively), where either the upstream or the downstream fragment is stabilised, but the other fragment is not, can serve to express co-transcribed genes differentially when the RNase Y cleavage allows the decay of either the upstream or downstream genes, thus combining maturation and decay-initiation in a single cleavage event. It is striking that none of the identified RNase Y cleavage sites generate downstream fragments that are destabilised in the ΔY strain, and only a single site (position 2436931, in the ncRNA srn_4470_RsaX28) generates an upstream fragment that is destabilised. Furthermore, only a single site (pos. 1620959 near *rpsT*) exhibits a decreased abundance of the downstream fragment in the ΔY mutant, making it the only site that might potentially be a transcription start site that has been misinterpreted as an RNase Y dependent cleavage site in the EMOTE data.

### RNase Y preferentially cleaves after guanosines

A consensus sequence for RNase Y cleavage was obtained by plotting the nucleotide frequency near the 99 identified RNase Y cleavage sites (Fig 2E). Two things stood out from this analysis: That RNase Y has a preference for cleaving immediately after a G (58%), and with lower frequency after A (30%), whereas only 10% and 2% of cleavage sites were after U and C, respectively, and that the region of cleavage has a tendency to be A/U-rich. Neither of these two rules appear to be strictly required, although the preference for G is especially striking in *S*. *aureus* where the genome is 67% A+T. It is thus likely that other factors such as secondary structures or protein partners are important for target selection. For example, the RNase Y cleavage site in the *sae*-operon identified by Marincola and coworkers [8], and re-identified in our EMOTE assay (SA0663 in Fig 1), is immediately after a U residue rather than a G or A (S2 Table).

To determine whether the primary sequence preferences of RNase Y are augmented by secondary structures, we defined three sub-regions of 50 nt in the vicinity of each RNase Y site: The 50 nt immediately upstream, the 50 nt spanning the cleavage site, and the 50 nt downstream of the cleavage (S6A Fig). Each sub-region was examined for potential secondary structures using the RNA folding program mFold [30] and then scoring the lowest possible ΔG for each sub-region. The median ΔG for all three sub-regions was between -3.9 and -5.8 kcal/mol, however the 50 nts spanning the cleavage sites consistently (in 93 out of 99 sites) had less predicted secondary structure than either the upstream or downstream regions (S6B Fig). This is not conclusive in itself, but consistent with previous observations that secondary structure in the vicinity of the RNase Y site will affect cleavage [4]. Nevertheless, it cannot be ruled out that large, multi-helix, secondary or tertiary structures such as pseudoknots are important for target site selection, but these are extremely hard to predict systematically *in silico*.

### Analyses of fragment fates in target RNAs

A set of transcripts were selected for further examination by Northern blot, both to confirm the RNase Y cleavage sites inferred by the transcriptome-wide data and to determine the lengths of the detected RNA, since neither RNA-seq nor EMOTE provide accurate information regarding the integrity of the RNA.

#### T-box riboswitches

**Fig 3:**
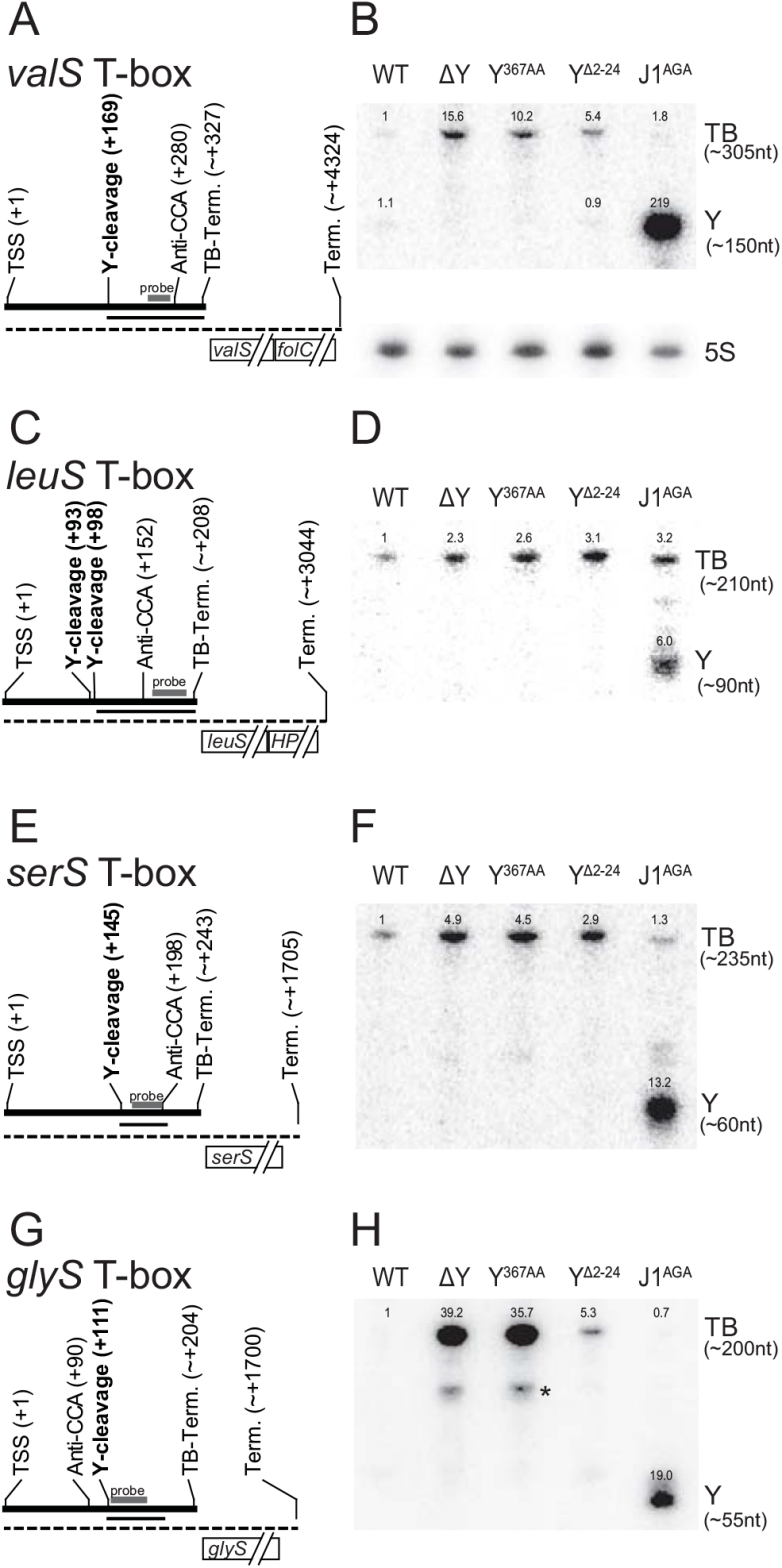
T-box riboswitches cleaved by RNase Y. (A) Layout of the *valS* T-box, indicating Transcription Start Site (TSS), RNase Y cleavage site (Y-cleavage), the highly conserved T-box motif that binds to the 3' CCA of uncharged tRNAs (Anti-CCA), and the predicted T-box riboswitch transcriptional terminator (TB-Term.). The thick line indicate the terminated T-box RNA, and the dotted thin line indicate the full-length transcript. The approximate extent of the fragment that appears in the J1^AGA^ mutant is shown with a thin line. The probe used for the Northern blot in panel B, is indicated with a thick grey bar. (B) Northern blot showing the terminated *glyS* T-box riboswitch (TB). A fragment downstream of the RNase Y cleavage site accumulates in the RNase J mutant (Y). Additionally, a sub-fragment of about 125 nt is detectable in the ΔY and Y^367AA^ strains (asterisk). Measured sizes of the detected fragments are indicated to the right. Relative intensities are indicated above each band. Loading was normalised to 5S rRNA (shown separately underneath), and the intensity of the band corresponding to the full-length T-box in WT lane set to 1. (C and D) The *leuS* T-box, with indications like panel A and B. (E and F). The *serS* T-box, with indications like panel A and B. (G and H) The *glyS* T-box, with indications like panel A and B.

From the list of putative RNase Y cleavage sites identified by EMOTE, it was striking that seven sites were located immediately upstream of aminoacyl-tRNA synthetases. This group of genes are often regulated by T-box riboswitches, which sense the level of tRNA molecules charged with amino acids, and terminate transcription prematurely if the level is already sufficient [31]. Four predicted T-box riboswitches were examined by Northern blotting: TB-valS, TB-leuS, TB-serS and TB-glyS. The prematurely terminated 5'-UTR was observed for all four T-boxes, and the accumulation observed by RNA-seq was confirmed for TB-glyS, TB-serS, and TB-valS (Fig 3). No degradation intermediates were observed to accumulate significantly in neither WT nor RNase Y mutant strains, presumably because degradation initiated by RNase Y is the rate-limiting step. Therefore, in order to observe the cleavage site identified by EMOTE, a mutant with the highly efficient 5' to 3' exoribonuclease J1 inactivated (Strain J1^AGA^) was used to prevent further processing of the downstream cleavage products generated by RNase Y. This approach revealed RNA fragments corresponding to the distance between the RNase Y cleavage and the riboswitch-induced transcription termination site for *valS* and *leuS* T-boxes (Fig 3B and 3D). In contrast, the downstream fragments that appear for the *glyS* and *serS* T-boxes are shorter than the distance between the RNase Y cleavage site and the T-box transcriptional terminator. The *glyS* T-box was therefore examined further, by using three additional probes to cover the entire 204 nt RNA. The two probes hybridising upstream of the RNase Y cleavage site were unable to detect the fragment that accumulates in J1^AGA^, demonstrating that it is indeed a result of cleavage at position +111, and is probably slightly shortened by 3' to 5' exonucleases until one of the many T-box secondary structures prevent further 3' processing (S7 Fig).

Although T-box riboswitches are expected to have secondary and tertiary structures that allow them to distinguish their respective substrates with high specificity, there is currently insufficient data to allow *in silico* structure-prediction of the regions where RNase Y cleaves the four T-boxes. The cleavage sites in TB-valS, TB-leuS and TB-serS are all in the region between the so-called “Stem I”, which recognises the anti-codon of the tRNA, and the highly conserved “T-box sequence”, which participates in either a terminator or an anti-terminator structure depending on whether the tRNA is charged [31]. In TB-glyS, the RNase Y cleavage site fall within a “T-box sequence” which is atypically elongated and thus precludes predictions about its structure.

#### *rpsB* and *tsf*

**Fig 4:**
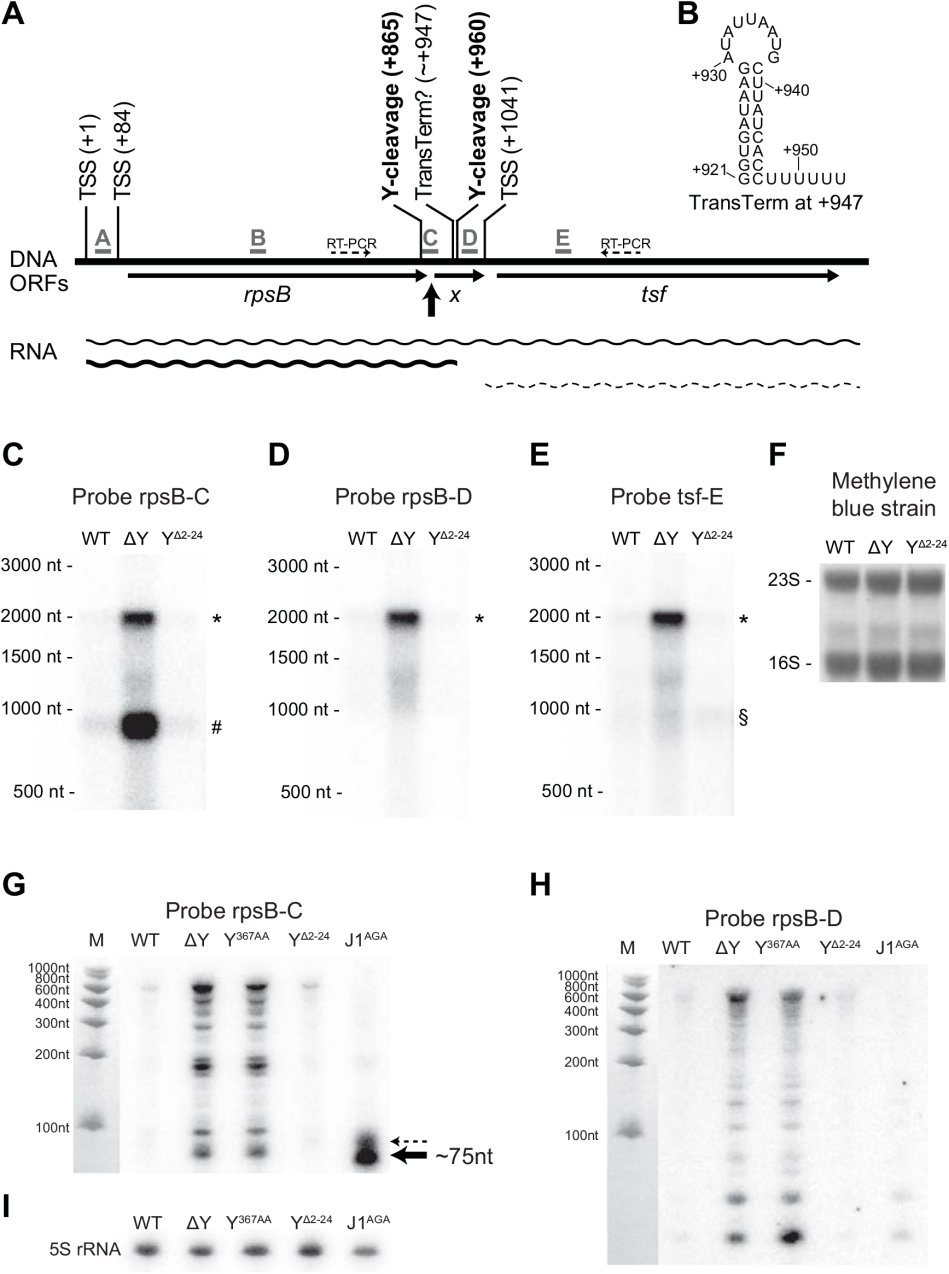
*rpsB* and *tsf* are co-transcribed via partial read-through of a transcriptional terminator, and both transcripts are stabilised in ΔY. (A) Overview of the *rpsB-tsf* locus, with the various predicted transcription start sites (TSSs) indicated. The five probes used for Northern blotting are shown in grey, and the location of the fragment detected with probe C in the J1^AGA^ mutant is highlighted by a thick black arrow. Thin arrows indicate open reading frames, including the short unannotated ORF *X*. RNase Y cleavage sites are marked in bold, and the question mark indicates the frequent read-through of the transcriptional terminator located inside ORF *X*. Thin dotted arrows indicate the amplification by RT-PCR (see S4 Fig). (B) Predicted hairpin structure of the transcriptional terminator at position +947. Panels C to F show the Northern blot membrane probed with rpsB-C, rpsB-D, tsf-E and stained with methylene blue as loading control. The read-through transcript (*), the *rpsB* transcript (#) and the *tsf* transcript (§) are indicated. Panels G to I show Northern blots optimised for short fragments (8% acryl-amide/urea gel) and probed with rpsB-C and rpsB-D, as well as a 5S-rRNA probe as loading control. In panel G, note the ~75 nt RNA that accumulate in the J1^AGA^ mutant (thick black arrow) and a minor band just above (thin dotted arrow).

The loss of RNase Y activity generates a dramatic effect for the *rpsB* and *tsf* ORFs (encoding ribosomal protein S2 and elongation factor TS, respectively), both in terms of half-life and steady-state levels (Fig 1, Tables 2 and S1). A predicted rho-independent transcriptional terminator can be identified between these two genes (Fig 4B and position +947 in Fig 4A; [32]), but previous studies, based on both microarray and RNA-seq data, suggest that they might be co-transcribed [32,33]. The identification, by EMOTE, of an RNase Y cleavage site inside the *rpsB* ORF (Fig 4A, position +865), suggests that RNase Y can initiate decay of the *rpsB* transcript, and thereby influence the stability, and consequently the steady-state level. However, no RNase Y sites were detected in the *tsf* transcript, and we therefore examined whether *rpsB* and *tsf* are indeed in an operon (i.e. whether there is read-through of the transcriptional terminator), and whether the steady-state levels of such a read-through transcript are affected by the deletion of RNase Y. Northern blotting with probe rpsB-C, which anneals immediately upstream of the transcriptional terminator (Fig 4A and 4C) revealed a major transcript of ~900 nt (corresponding to termination at the terminator) and a slightly weaker signal at ~2000 nt (corresponding to read-through of the terminator). As expected from the RNA-seq, both of these transcripts were massively accumulated in the ΔY strain (Fig 4C), indicating that the stabilisation of *rpsB* and *tsf* observed in the rifampicin assay (Fig 1) is the cause, although it cannot be ruled out that the ΔY mutant exhibits increased *rpsB* promoter activity, which would also contribute to the increase in steady state level. A probe annealing inside the *tsf* ORF (Fig 4E), also gave rise to two bands in the WT strain: a ~2000 nt transcript, corresponding to the read-through from the *rpsB* promoter, and a ~1000 nt RNA which would correspond to a transcript from the predicted *tsf* promoter (Fig 4A, position +1041). In the ΔY strain, the signal for the ~2000 nt band is much stronger than for the ~1000 nt band, indicating that whatever affects the *tsf*-encoding RNA must happen upstream of *tsf* transcription start site. Probe rpsB-D hybridises between the transcriptional terminator and the *tsf* transcription start site, and detected neither the ~900 nt band from probe rpsB-C nor the ~1000 nt band from probe tsf-E, verifying that these transcripts do not overlap (Fig 4D).

In order to examine the finer details of RNase Y cleavage, a Northern blot with higher resolution was prepared (8% acryl-amide urea gel), and lanes with Y^367AA^ and J1^AGA^ mutants were included. The upstream fragment generated by RNase Y cleavage at +865 is never detected (S8A and S8B Fig), and it might be rapidly degraded by 3’ exonucleases. However, a small RNA fragment that is normally removed 5' to 3' exonucleolytically by RNase J (thick black arrows in Fig 4A and 4G) appears when the J1^AGA^ strain is hybridized with probe rpsB-C. The major band probably corresponds to RNase Y dependent cleavage of the *rpsB* transcript terminated at ~+947, and a minor RNA species, indicated by a dotted arrow in Fig 4G, may correspond to cleavage or the read-through transcript which as then been cleaved a second time by RNase Y at position +960, however, the resolution of the Northern blot is not high enough to be certain.

Northern blotting of the *rpsB* gene revealed that a large number of sub-fragments accumulate in ΔY and Y^367AA^ mutants (Figs 4 and S8), but are only observed in WT, Y^Δ2–24^ and J1^AGA^ when over-exposing the blot. The longest of the fragments are only about 600 nt, which indicates that they are degradation products, and since they abound in the ΔY and Y^367AA^ mutants, they must generated in an RNase Y independent manner.

### The RNase Y membrane anchor is not essential for endoribonuclease activity

Whatever the evolutionary explanation for the anchoring of RNase Y to the membrane, it has the consequence that only RNA molecules that are close to the membrane can be cleaved. RNase E in *E*. *coli* is also anchored to the membrane, albeit by a completely different type of anchor (an amphipatic helix) [34], and it therefore seems likely that membrane association is generally advantageous for bacterial RNA degradation machinery. While both ΔY and Y^367AA^ mutants exhibit growth-curves that are very similar to the WT strain, at 37°C in Mueller-Hinton broth, the anchorless Y^Δ2–24^ mutant grows significantly slower, and thereby indicates an important function of the membrane anchor that is dissimilar to the loss of enzymatic activity in the ΔY and Y^367AA^ mutants (S3 Fig).

There are at least three potential functions of a membrane anchor, activity enhancement, activity limitation, and/or sequestering another protein.

1) Is the membrane anchor needed for the endoribonucleolytic activity of RNase Y?

**Fig 5:**
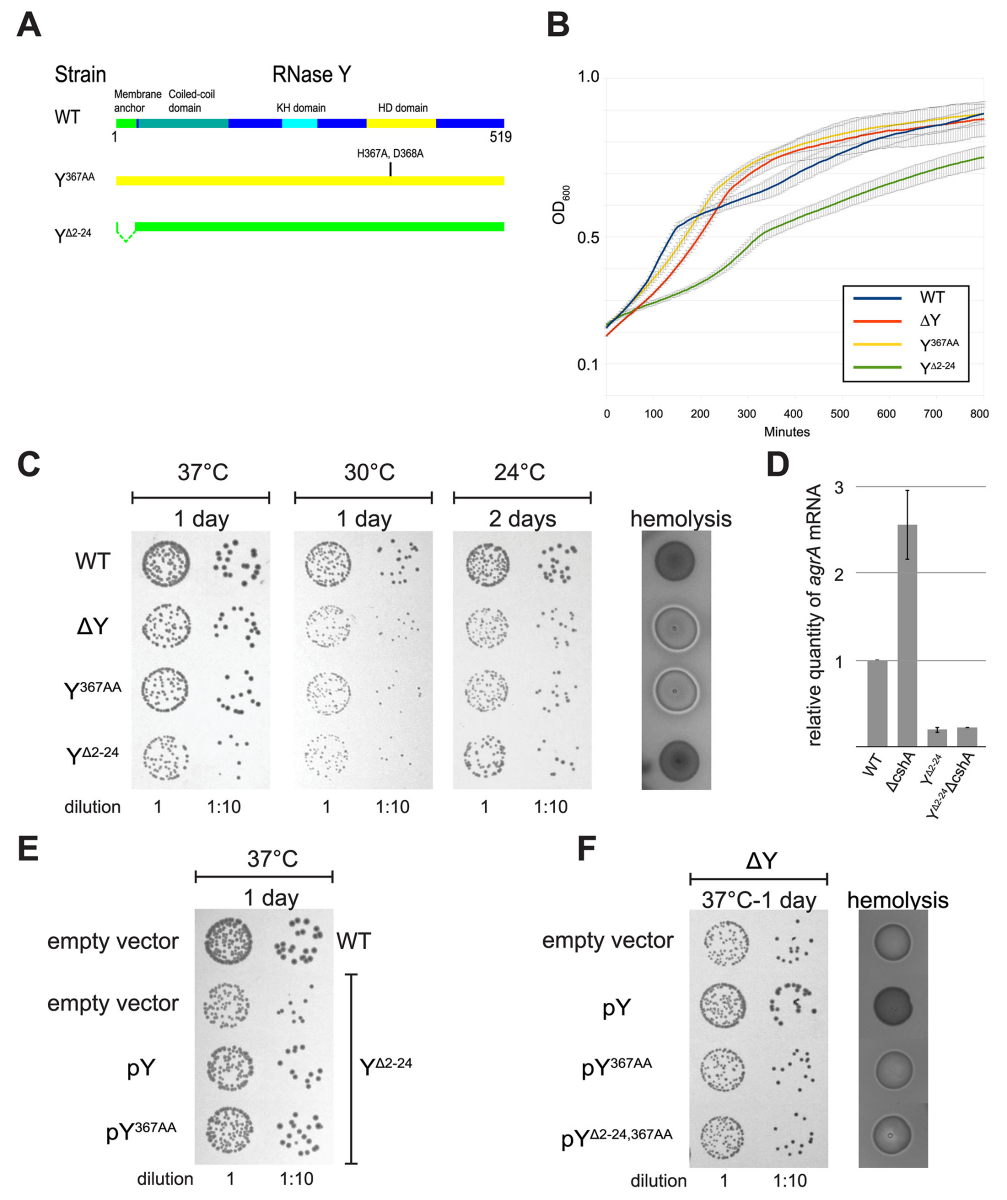
Removal of the membrane anchor enables RNase Y to suppress the phenotypes of a ΔcshA mutant. (A) In the left panel, over-night cultures of single mutants ΔcshA, ΔY and Y^Δ2–24^ and double mutants ΔYΔcshA and Y^Δ2–24^ΔcshA were diluted, spotted on agar-plates, and incubated at the indicated temperatures and times. In the right panel, over-night cultures were spotted on horse-blood-agar. (B) Transformants of strain ΔYΔcshA with plasmids expressing variants of RNase Y were selected at 42°C, then restreaked and grown over night at 42°C. Finally the cultures were diluted and spotted at the indicated temperatures. ΔYΔcshA with pY^Δ2–24^ grows significantly better than the other strains at 24°C. (C) Cartoon showing the four versions of RNase Y expressed from the plasmids; wild-type RNase Y (pY), anchorless RNase Y (pY^Δ2–24^), RNase Y active site mutant (pY^367AA^), and anchorless RNase Y active site mutant (pY^Δ2–24,367AA^). (D) The Y^Δ2–24^ΔcshA strain was transformed with the plasmids expressing the wild-type RNase Y, Y^367AA^ or Y^Δ2–24,367AA^. Overnight cultures were diluted, spotted on agar-plates and incubated at the indicated temperatures for the indicated period of time. Both pY and pY^367AA^ inhibit growth at 24°C. (E) Cartoon showing how the wild-type RNase Y and Y^367AA^ can anchor the Y^Δ2–24^ protein back to the membrane, via dimer-formation.

The Y^Δ2–24^ mutant was examined by EMOTE, and it became immediately apparent that the membrane anchor is not a requirement for RNase Y enzymatic activity. Every one of the 99 identified RNase Y cleavage sites exhibited a signal in the Y^Δ2–24^ EMOTE data-set (S3 Table), providing evidence that the Y^Δ2–24^ protein is capable of cleaving the same sites as wild-type RNase Y, and in contrast to the ΔY mutant, the hemolysis of the Y^Δ2–24^ mutant is not enhanced (Fig 5A), and the *agr* mRNA levels are actually decreased (S3D Fig).

2) Is the membrane anchor needed to sequester RNase Y to the membrane in order to prevent its endoribonucleolytic activity from cleaving indiscriminately?

If RNase Y is membrane-confined in order to limit its own activity, then removing the N-terminal membrane anchor should lead to a decrease in half-life for RNA molecules that are normally protected by their mid-cytosolic location. However, few additional cleavage sites where identified in the EMOTE data of Y^Δ2–24^ (S2 Table), and it is possible that the Y^Δ2–24^ enzyme does not gain additional cleavage sites, but instead cuts existing sites too vigorously. This seems to be the case for the *agr* mRNA, which is much less abundant in Y^Δ2–24^ than in the wild-type strain (S3D Fig), presumably due to an effect that is opposite to the stabilisation seen in the ΔY mutant (Fig 1). However, the EMOTE data for strain Y^Δ2–24^ does not immediately reveal an increased activity, since such an effect can be accompanied by changes in expression levels or degradation by other RNases.

3) Is the membrane anchor needed to anchor a protein partner of RNase Y to the membrane?

Both strain ΔY and Y^367AA^ have lost RNase Y activity, but whereas ΔY can neither bind other proteins nor anchor them to the membrane, the Y^367AA^ mutation presumably only affect the enzymatic activity of RNase Y itself, with no change in the intra-cellular localisation. The overall correlation between the EMOTE data of ΔY and Y^367AA^ is very strong (Pearson coefficient of 0.95; S2 Table). Therefore, if RNase Y serves as an anchor for a protein partner which needs to be membrane associated, then such a partner does not appear to affect the 5'-ends of the RNA.

### Removal of the membrane anchor enables RNase Y to suppress the phenotypes of a Δ*cshA* mutant

Similar to what was previously observed for a *cshA* deletion strain (ΔcshA), the *agr* mRNA is stabilised in the ΔY mutant (Fig 1), leading to an increase in hemolysis on horse-blood agar-plates. In contrast, the Y^Δ2–24^ strain show neither increase in *agr* levels nor in hemolysis compared to wild-type, indicating that the anchorless RNase Y protein is still able to target the *agr* mRNA, and indeed may do so with high efficiency (S3D Fig). When a *cshA* deletion was generated on the chromosome of the ΔY mutant, the resulting strain (ΔYΔcshA) was phenotypically highly similar to ΔcshA, with reduced growth at 24°C and increased hemolysis (Fig 5A).

Surprisingly, the same Δ*cshA* mutation in combination with the Y^Δ2–24^ mutation (Y^Δ2–24^ΔcshA) was not only able to grow at low temperature but additionally had wild-type levels of hemolysis (Fig 5A), despite the fact that the Y^Δ2–24^ strain itself grows poorly (S3 Fig).

This novel anchor specific phenotype–i.e. the suppression of Δ*cshA* phenotypes–was used to further examine the role of the membrane anchoring. Plasmids encoding wild-type, active-site mutated and anchorless active-site mutated RNase Y were readily transformed (pY, pY^367AA^ and pY^Δ2–24,367AA^, respectively) (Figs 5B, S3D and S3E). In contrast, several unsuccessful attempts were made to transform a vector over-expressing anchorless RNase Y (pY^Δ2–24^) into our various *S*. *aureus* mutants, leading to the conclusion that such a construct is toxic to the cell, consistent with the slow growth observed for the chromosomal Y^Δ2–24^ mutant, caused by just a single copy of the mutated gene (S3 Fig). However, we hypothesised that it might be possible to transform the pY^Δ2–24^ vector into the ΔYΔcshA strain at 24°C, since at this temperature, a single copy of the anchorless RNase Y gene actually enhances growth of a Δ*cshA* mutant, by suppressing the cold-sensitive phenotype (Fig 5A). Indeed, transformant colonies of ΔYΔcshA *+* pY^Δ2–24^ did appear on the agar-plates incubated at 24°C, in contrast to the parallel controls plated at 30°C and 37°C. Unexpectedly, transformation at 42°C, the third control temperature, also resulted in viable, albeit small, colonies on selective plates.

These ΔYΔcshA *+* pY^Δ2–24^ cells isolated at 42°C–which have never been cultivated at 24°C, and thus have therefore never been subjected to selective pressure to accumulate additional compensatory mutations that would allow growth at 24°C–enhanced growth of ΔYΔcshA *+* pY^Δ2–24^ at 24°C, compared to ΔYΔcshA *+* pY^367AA, Δ2–24^, pY and pEB01 (empty vector). This proves that the presence of enzymatically active anchorless RNase Y directly suppresses the cold-sensitivity of the Δ*cshA* mutant (Fig 5B).

Is it really the membrane-confinement that prevents a wild-type RNase Y from negating the Δ*cshA* phenotypes? To address this question, the Y^Δ2–24^ΔcshA strain was transformed with a multicopy plasmid expressing wild-type RNase Y (pY), thereby attempting a re-anchoring of the anchorless RNase Y proteins to the membrane, via dimerisation with the wild-type RNase Y (mediated by the coiled coil domains, which can dimerise without the presence of the membrane anchor). The wild-type protein is presumably translated at the membrane whereas the anchorless RNase Y would have no such constrictions, stacking the cards against hetero-dimers, and the re-anchoring strategy was therefore not expected to be 100%. Nevertheless, a partial reversion to the cold-sensitivity was clearly observed, both with the pY plasmid, and with an equivalent vector expressing an active site mutant of RNase Y (pY^367AA^). In contrast, an anchorless version of the latter (pY^367AA, Δ2–24^) was unable to revert the suppressed Δ*cshA* phenotype (Fig 5D).

## Discussion

RNA cleavage, maturation and decay are crucial steps in gene expression in any living cell. Our data indicate that RNase Y does not cleave all transcripts pervasively, the way RNase E has recently been shown to do in *E*. *coli* [35]. The genes where the rate-limiting degradation step can be confidently assigned to RNase Y number less than a hundred, and control mechanisms must therefore be in place to regulate RNase Y activity. We have here explored how the membrane-anchor reduced access to the substrates, as well as how primary sequence preferences and perhaps also secondary structures might limit/modify activity once an RNA substrate is encountered.

### RNase Y-dependent decay-initiation and cleavage specificity

We have here presented for the first time a transcriptome-wide mapping of *in vivo* generated endoribonucleolytic cleavage sites that reveals the sequence preference of a decay-initiating RNase (Fig 2E). This data has been correlated with RNA stability measured on a global scale, confirming a link between RNase Y cleavage and the rate-limiting steps of RNA degradation (Fig 1).

The cleavage sites are identified by EMOTE as they appear *in vivo*, ensuring the presence of any secondary factors (either nucleotide or protein) that might be absent in an *in vitro* assay, and the precise manner by which the sites are mapped uncovers even single-nucleotide site-preferences. The observed preference for cleaving after G cannot be an artefact of the EMOTE assay, because the method detects the downstream fragment, which indeed might potentially be biased by sequence preference in either the ligation, reverse transcription, amplification, or sequencing steps. However, the G is located in the upstream fragment, which is inferred from the genome sequence and is therefore not detected directly by EMOTE.

Importantly, the method reveals a class of RNase Y sites that could never be discovered with classic RNA-seq methods because neither steady state nor stability is altered (central part of Fig 1). This sub-population of “stability-neutral” RNase Y sites includes the maturation site in the *sae* operon, previously identified by Marincola and coworkers, and we presume that many more of these stability-neutral sites are indeed maturation cleavage events. However, many of the identified stability-neutral RNase Y sites fall within open reading frames, and these cleavage events, although they do not alter the abundance or stability of the transcripts, will certainly prevent protein-expression from the cleaved ORFs, and have strong potential to be a regulatory mechanism (Fig 1, red Xs in the central part).

RNase Y cleavage sites were identified in or near many of the stabilised ORFs, however 74 stabilised ORFs did not have a corresponding identified RNase Y site. This could be due to indirect effects on stability, for example via regulation of other factors in RNA degradation (see below), or could reflect the limited number of RNase Y sites identified with our strict criteria.

Cleavage sites in transcripts encoding the RNA decay machinery suggests self-regulation:

It was recently discovered that the *S*. *aureus* RNase III cleaves its own transcript, thereby self-regulating its own expression level [36]. The presented list of RNase Y cleavage sites includes several of the mRNAs encoding either RNases or their protein partners (Table 2, Fig 2), suggesting a possible regulatory network, where the abundance (or activity) of RNase Y determines the level of itself and other enzymes in the RNA degradation machinery.

Is there such a regulatory system? The cleavage sites within the coding region of the RNase J2 and phosphofructokinase mRNAs will certainly prevent protein expression. The cleavage sites in the *SA0941-rnjA* and *cvfA* operons are all in the 5’ UTR, close to the transcription start sites, and do not directly interrupt the ORFs (Fig 2), but it is possible that RNase Y cleavage either induces or prevents formation of secondary structures that modify how much these two mRNAs are translated.

### RNase Y cleavage of ncRNAs

Ten of the detected RNase Y cleavage sites fell within annotated ncRNAs (S2 Table), five of these into T-boxes (Fig 3), and three within 5’-UTRs of ORFs that either accumulate or are stabilised in the ΔY mutant (*fer*, *valS* and *glyS*; S1 Table and S4C Fig). It is even possible that it is an RNase Y cleavage that defines the 5’-end of srn_4240_Teg84 and the 3’-end of srn_1380_Teg46, since the cleavage sites are 1 and 2 nucleotides from the annotated ends, respectively (S2 Table). These numbers are probably an under-estimation of the real impact of RNase Y upon ncRNAs, since the way the EMOTE assay was performed, both at the RNA isolation step and the size-selection step (S2 Fig) preferentially detects downstream cleavage fragments that are longer than about 160 nt, and thus may easily miss cleavage sites in short RNAs, but readily detects the cleavage site in the 1177 nt srn_4470_RsaX28 (S1 and S2 Tables). ncRNAs often have elaborate secondary structures, which might influence (or target RNase Y to) the cleavage site, and while none of the cleaved ncRNA have a known structure, the four T-box riboswitches do have characterised features. The RNase Y cleavage sites for three of them (TB-valS, TB-serS and TB-leuS) fall in the “variable region” between the so-called “Stem I” and the anti-CCA [31], and for the *glyS* T-box, the RNase Y cleavage falls within the exceptionally long distance (about 40 nt) separating the anti-CCA part of the T-box sequence and the terminator/anti-terminator part of this T-box (Fig 3). Unfortunately, these regions are exactly those where the local structure cannot be predicted, especially when tRNA is bound to the riboswitches, and although it seems almost certain that secondary structures affect RNase Y cleavage and target selection, it is still uncertain how this comes about.

### RNase Y initiated RNA decay, one of several parallel degradation pathways

Only about a hundred ORFs have significantly increased half-lives in the ΔY mutant (Table 1), and it is therefore clear that RNA degradation mechanism(s) must exist that are independent of RNase Y.

The overall mRNA level of *rpsB* is clearly augmented in the RNA-seq of the ΔY mutant, however this does not necessarily reflect an increase in the potential for translating the encoded S2 ribosomal protein, since the short sub-fragments discovered in Figs 4G, 4H and S8. clearly contribute to abundance measured by global methods such as RNA-seq and micro-arrays, but also non-global RNA quantification methods such as qRT-PCR. Only Northern blots can show that intact *rpsB* RNA truly accumulates in the ΔY mutant (Fig 4C).

The small *rpsB* fragments that are readily visible in the ΔY and Y^367AA^ mutants are actually also generated in the WT, Y^Δ2–24^ and J1^AGA^ strains (S8 Fig), although some of them are only readily detected when the Northern blot is over-exposed. We therefore propose that in a normal wild-type cell, there are two parallel degradation pathways for the *rpsB* RNA, each with their own rate-limiting step (S8G Fig): The major pathway is initiated by RNase Y cleavage at position +865, which generates two fragments; the downstream fragment (black arrow in Fig 4G) is rapidly degraded by the 5'-exoribonucleolytic activity of the RNase J1/J2 complex, whereas the upstream fragment is equally rapidly degraded by one or more of the 3' exoribonucleases. In the minor “alternative” pathway, an unknown endoribonuclease cleaves at multiple locations inside the *rpsB* ORF, generating the pattern of fragments observed in S8 Fig. Once the RNase Y mediated decay pathway is removed, then the *rpsB* RNA accumulates and so does the intermediate products of the inefficient minor decay pathway. In support of this hypothesis, a similar pattern of sub-fragments was observed when Northern blotting was used to examine the *rplS* gene, encoding the L19 ribosomal protein (S9 Fig).

### RNase Y, the membrane-anchor and CshA

The transcriptome stability effect of a Δ*cshA* mutation was recently examined in a strain that is otherwise isogenic to the mutants used in this study [19], and re-analysing that data using the Smallest-difference model used in the present study, revealed that only four transcripts are stabilised in both ΔY and ΔcshA (*SA0964*, *recU-pbp2*, *SA1970* and *gltT*). Such a small overlap does not mean that CshA and RNase Y never work together, but it does mean that each of the these two RNA decay factors comprise the rate-limiting step for a different set of RNA molecules, and thus participate in separate RNA degradation pathways.

It is therefore not immediately clear why the Y^Δ2–24^ mutation rescues the Δ*cshA* phenotypes so efficiently (Fig 5). It seems logical that it is an RNA stability/steady-state effect, which is supported by the reduced level of *agr* mRNA in the Y^Δ2–24^ mutant (S3D Fig), and it might only be the levels of a single Δ*cshA*-stabilised RNA that needs to be adjusted. However it is also possible that these phenotypes are unrelated to RNA decay, since other bacterial DEAD-box RNA helicases are involved in processes like ribosomal maturation or translational regulation (reviewed in [37]). Inspection of the Northern blots in Figs 3, 4, S7, S8 and S9, shows that anchorless RNase Y exhibit a profile identical to the WT strain in some cases (*rpsB* and *rplS*), but looks more like an intermediate between WT and ΔY for the T-box riboswitches, with a clear increase in abundance of the full-length T-box RNA (Figs 3 and S7). The evolutionary reason for anchoring an endoribonuclease to the membrane is therefore far from straight-forward, although its importance can be seen in both this *S*. *aureus* study and in previous studies of the membrane-anchoring amphipatic helix of RNase E in *E*. *coli* [34].

### Conclusion

We present a study of the activity of RNase Y in *S*. *aureus* where we integrate global data from several methods in order to pinpoint specific *in vivo* activities of the enzyme: We have identified a preferred target sequence (a guanosine immediately prior to the cleavage site) and we have examined which RNA molecules are stabilised in an RNase Y mutant.

Since all RNAs have guanosines, it is difficult to imagine how this preference can be the sole criteria for selecting specific RNA molecules for degradation. However, if a guanosine is found in combination with the right adjacent secondary structure, as seem to be the case for the T-box RNAs, then that could augment the specificity considerably. Finally, the sub-cellular location of RNase Y certainly restricts the target choice, and we show that lifting this requirement in itself (in the Y^Δ2–24^ mutant), leads to severe difficulties for the cell. Despite this, the Y^Δ2–24^ mutation is able to suppress the cold-sensitive phenotype caused by the deletion of the CshA RNA helicase, which presumably generates a global increase in secondary RNA structures that inhibit exoribonucleases, and we speculate that RNase Y, once liberated from the membrane, is able to provide new access-points for the exoribonucleases and relieve the burden of mis-regulated RNA decay.

## Materials and Methods

### Growth and RNA isolation

All strains were grown in Mueller-Hinton broth (Becton-Dickinson) with uracil (20 mg/l) and appropriate antibiotics at the following concentrations: 10 mg/l chloramphenicol, 10 mg/l erythromycin, 50 mg/l kanamycin.

For RNA isolation, cultures were harvested in mid-exponential phase at an OD_600_ of ~0.4, and RNA was purified using either RNeasy Mini Kit (Qiagen) for RNA-seq and EMOTE, or Trizol (Ambion) for Northern blots (see Supplemental Experimental Procedures).

### Decay and steady-state analyses

Quantification, normalization, and decay half-lives were computed for each assay following the procedure described in [19]. Briefly, reads are mapped to the *S*. *aureus* N315 genome with the software BWA [38]. A script in the R programming language, counted the number of them that overlaps a gene, and discarded those that align to multiple positions on the genome or on the inappropriate strand. A normalization step further adjusted genes counts according to the level of expression of the housekeeping gene *HU*, which has a very long half-life. Half-lives were then obtained by fitting a weighted linear model on log2-transformed expression values of each gene.

Quality of the half-life estimates were asserted by checking for a minimum read count of 100, on average over the four time points, and a minimum value of 20 on the fitted error of the linear models. Additionally, duplicated experiments of the WT assay and the ΔY assay were compared pairwise in a smallest difference model to determine the most conservative fold change in half-life and in steady state level for each gene.

### EMOTE assay

Approximately 4 μg total RNA from each sample was used for the EMOTE assay (see details in Supplemental Experimental Procedures, and S2 Fig), the full data-sets are given in S3 and S4 Tables.

For each potential RNase Y cleavage site, the following very stringent criteria were used to ensure a high level of confidence for the identified RNase Y cleavage sites:

1. Only unambiguously mapped reads are considered–This avoids counting reads twice, and at the same time eliminates ribosomal RNA from analyses.
2. For each sample, the Quantification Sequence Counts of 5'-ends detected by EMOTE are normalized by dividing with the total number of unambiguously mapping reads (more than 600000 for all samples; S2 Table) from the sample and then multiplied by 1000000 –This allows direct comparison between EMOTE data from different RNA samples and sequencing runs.
3. This Normalised EMOTE Quantification Count (NEQC) of the WT strain is required to be higher than 10 –Thus in each biological replicate, the same 5'-end is detected independently at least 10 times per million reads.
4. The NEQC of both the ΔY and Y^367AA^ strains are required to be 5 times less than the quantification of the WT–This selects for 5'-ends that are generated by RNase Y.
5. Constraints 3) and 4) are required for both duplicates of the EMOTE assays.
6. The number of reads from the RNA-seq of the ΔY strain that maps to a window of 300 nt surrounding the position must not be less than 2 fold lower than the number of reads in the RNA-seq data from the WT strain–This is to avoid false positives when an RNA is not expressed in the ΔY strain, which would otherwise automatically qualify all 5'-ends within this RNA with respect to constraint 4).
7. The absolute number of ΔY RNA-seq reads mapping to the 300 nt window must be larger than 200 in both biological replicates–This is to ensure that the data in 6) is within the measurable range.
8. If two or more immediately adjacent positions conform to the above criteria, then the site with the highest number of independently detected 5'-ends in the WT EMOTE data will be taken, and neighbouring positions discarded–This is to avoid bias in the following analyses, in the event that RNase Y cleaves slightly imprecisely.

### Northern blots

Separation of large RNA molecules: 4 μg total RNA was loaded in each lane of a 1.5% agarose gel with formamide and MOPS buffer. After electrophoresis, the RNA was transferred to a Hybond-N membrane (Amersham) by capillary blotting, UV crosslinked, and the marker as well as ribosomal RNA were visualised with methylene blue

Separation of small RNA molecules: 5 μg total RNA was loaded in each lane of an 8% acryl-amide gel with 8M urea. After electrophoresis, the RNA was transferred to a Hybond-N membrane (Amersham) by electroblotting, UV crosslinked, and the marker was visualised with methylene blue.

The ^32^P labelled synthetic DNA probes were detected with a Typhoon FLA7000 phosphoimager (General Electric). The membrane was stripped and re-used multiple times, each time verifying the stripping by phosphoimager (see Supplemental Experimental Procedures).

### Accession numbers

The full data sets from EMOTE and RNA-sequencing have been deposited in the GEO database with accession numbers GSE68811.

## Supporting Information

S1 Text Supporting Materials and Methods. (PDF)

S1 Fig Non-coding RNA (ncRNAs) steady-state levels and stability are affected by RNase Y. The change between WT and ΔY in the half-life of each ncRNA was estimated using a smallest-difference model, and the same was done for the steady-state levels of the ncRNAs. The fold-change in half-life is plotted on the x-axis and the fold-change in steady-state level on the y-axis. Each grey dot represents an ncRNA transcript where sufficient data was available (67 in all). Red X's show which ncRNAs contain an identified RNase Y cleavage. The list of ncRNAs was based on the database from Sassi et al. (2015).
(PDF)

S2 Fig Flowchart of the EMOTE method. RNA inside the cells are a mixture of tri-phosphorylated (primary) transcripts, and mono-phosphorylated (processed) transcripts. The 5'-bases of the mono-phosphorylated RNAs are marked with asterisks, and the downstream fragment generated by an RNase Y cleavage is in red (absent from the ΔY strain). The two total RNA samples, one from the WT strain and one from the ΔY mutant are separately ligated to the Rp6 oligo (light blue), a reaction that only works for 5' mono-phosphorylated RNA. The Rp6 RNA oligo has 7 random bases, the so-called Quantification Sequence, in the middle (indicated by NN). The ligated RNA is reverse transcribed with a primer which has a random sequence near the 3' end, and a tag (light green) at the 5' end. This generates cDNA with a known sequence at the 5' end. At the 3' end, a cDNA molecule will also have a known sequence, if (and only if) the reversely transcribed RNA molecule was ligated to the Rp6 oligo (i.e. the RNA molecule was mono-phosphorylated in the cell). The cDNA that has the cRp6 tag at one end and the reverse transcription tag at the other end is then PCR-amplified with primers that add the appropriate adaptor-sequences for Illumina sequencing at the ends (Illumina 1.0 and Illumina 2.0, marked in dark blue and dark green respectively). Additionally, the primers that hybridises to the cRp6 sequence contain an EMOTE barcode (A and B) that is unique for each RNA sample. Once the EMOTE barcode has been added, then the PCR products can be mixed, since each and every molecule can be bioinformatically traced back to the original RNA sample it came from. The mix of PCR-products is then sequenced, using Illumina technology, from the “Rp6-end”. As little as 50 nt sequence will read through the EMOTE barcode and Quantification Sequence, into the first 20 nt of the original mono-phosphorylated RNA molecule. These 20 nt of transcriptome sequence, referred to as the Mapping Sequence, can be aligned to the place on the genome that corresponds to the 5' end of the original RNA molecule, and the exact chromosomal position of the 5' base (asterisk) can easily be determined. Since PCR is being employed in the EMOTE protocol, it is important to verify that reads mapping to the same position, actually reflect individual original RNA molecules. Within the EMOTE reads, if the 7 bases of Quantification Sequence are different, then the reads must originate from separate ligation events with different Rp6 molecules, and as a consequence, from different original RNA molecules.When millions of reads are sorted into their respective RNA samples, and mapped onto their respective chromosomal positions, it is possible to identify specific species of RNA 5' ends that are present in the WT data, but absent from the ΔY data. The positions of these 5' ends are good candidates for RNase Y cleavage sites (in red).
(PDF)

S3 Fig Growth and hemolysis of the RNase Y mutants with and without complementation. A) Overview of the RNase Y protein. The functional domains are according to Kaito et al. 2005 and Lehnik-Habrink et al. 2011: Membrane anchor, KH: RNA-binding domain, HD: Catalytic domain with the key HD motif, His367 and Asp368, that are mutated to alanines in the Y^367AA^ mutant. B) Growth curves of WT and RNase Y mutant strains. Growth of strains was monitored continuously, in quadruplicate, in a plate-reader at 37°C. Error-bars indicate standard deviation. The cultures were started from exponentially growing cultures, however a parallel experiment started from stationary cultures gave essentially identical results. C) Growth of RNase Y variants on agar-plate. Left panel: over-night cultures grown at 37°C, were diluted and spotted on MH-agar plate at the indicated temperature for the indicated time. On agar-plate, the ΔY strain grows slightly slower than the WT strain at 37°C. The difference in growth between WT and ΔY is more pronounced at low temperature. Both ΔY and Y^367AA^ grew equally whatever the growth condition indicating that the main effect observed with ΔY is due to its enzymatic activity as opposed to an indirect role through the binding of protein-partners. The Y^Δ2–24^ mutant as observed in liquid culture (B) grow markedly slower at 37°C than WT and ΔY strain. Our RNA-seq analyses (Fig 1) showed that the quorum-sensing *agr* mRNA both accumulate and is stabilised in absence of ΔY, leading to increase production of hemolysin, which are controlled by the *agr* system as shown in the right panel where the strains were plated on horse-blood agar. Both ΔY and Y^367AA^ spot have a similar halo of hemolysis, in contrast the Y^Δ2–24^ does not induce the production of hemolysin, indicating that the Y^Δ2–24^, still targets the *agr* messenger. D) qRT-PCR quantification of *agrA* RNA in mutants, relative to WT levels. All cultures were harvested at OD_600_ = 0.4, and the relative levels were normalised to mRNA of the *HU* housekeeping gene. Error-bars indicate the SEM. E) Growth of the Y^Δ2–24^ strain is restored in presence of the full length RNase Y protein. The Y^Δ2–24^ strain, transformed with the plasmids expressing RNase Y variants or the empty vector, was cultivated over-night at 37°C, and spot after dilution on a MH agar-plate at 37°C. The slow growth of the Y^Δ2–24^ mutant is suppressed when over-expressing the full-length protein, irrespective of whether the latter is active or not, suggesting that the full length proteins can re-anchor the anchorless RNase Y back to the membrane via dimerisation. F) Complementation assay in a ΔY strain by plasmids expressing the RNase Y variants. The ΔY strains, transformed with the plasmids expressing Y variants or the empty vector, were spotted on MH agar-plate (left panel) and horse-blood plate (right panel). pY complement both the growth and the hemolysis defect of a ΔY strain, showing that it is fully active and that the strep-flag-his tag fused at the C-terminal in these constructions do not seem to alter the RNase Y activity. As expected, the two phenotypes were not restored when the active site is mutated in the full-length (pY^367AA^) or the anchorless (Y^Δ2–24,367AA^) protein.
(PDF)

S4 Fig RNase Y cleavage sites and ORFs are on the same RNA molecules. (A) List of RNase Y cleavage sites that are close enough to an ORF to prove their connection via analyses of the RNA-seq data to find 50 nt Illumina reads that span the gap (number of spanning reads shown in the last column). (B) List of RNase Y cleavage sites that fall near ORFs, and where RT-PCR was performed to demonstrate that the cleavage site and the ORF are on the same RNA molecule. Sometimes two ORFs could be connected (two ORF-names in second column), and the distance to each ORF is shown in the final column. (C) Agarose gels showing the RT-PCR product used to define (B), with each reaction performed either without or with the reverse transcriptase step. Primer sequences can be found in the oligonucleotide table.
(PDF)

S5 Fig RNA immediately adjacent to RNase Y cleavage sites is often, but not always, stabilised in the ΔY mutant. 300 nucleotides upstream and 300 nucleotides downstream of the 99 identified RNase Y cleavage sites were examined for stability in the RNAseq data, and plotted in a scatter-plot. The majority exhibits no significant stability change, however around 20 sites cleavage events clearly influence the stability of upstream and/or downstream RNA. Class 1) If both upstream and downstream fragments were stabilised by the lack of cleavage (i.e. in the ΔY mutant), then the RNase Y cleavage is presumably an initiating event for decay of the entire RNA. Class 2) If neither the stability of the upstream, nor the stability of the downstream fragment are affected by the cleavage, then the cleavage probably represents a maturation event, but may still be regulatory if the cleavage site is situated inside an ORF. Class 3) and Class 4) If either the upstream or the downstream fragment is stabilised, but the other fragment is not, then the RNase Y cleavage can serve to express co-transcribed genes differentially, by allowing the decay of either the upstream or downstream genes, thus combining maturation and decay-initiation in a single cleavage event. Thus there are multiple outcomes of RNase Y endoribonucleolytic activity.
(PDF)

S6 Fig Predicted free energy of RNA folding near the RNase Y sites. A) The sub-sequences used for calculating predicted secondary structures near the 99 RNase Y cleavage sites. 50 nt upstream, 50 nt overlapping and 50 nt downstream of the sites. B) Box-plot showing the potential free energy of folding in the three sub-regions (dark grey). One sub-region of 50 nt upstream of the site (Upstream), one region of 50 nt straddling across the site (Overlapping), and one region of 50 nt downstream of the cleavage site (Downstream). As control, the sequences for each sub-region were scrambled, and the free energy was calculated (light grey). The thick black line shows the median, the grey boxes the 25% and 75% quartiles and the error-bars are an estimate of the 95% confidence interval. Dots show the position of individual outliers.
(PDF)

S7 Fig Detailed Northern blot analyses of the *glyS* T-box degradation products. A) Northern blot with probe glyS-TB-A. B) Northern blot with probe glyS-TB-B. C) Northern blot with probe glyS-TB-C (Same exposure is shown in Fig 5H). D) Northern blot with probe glyS-TB-D. E) 5S rRNA loading control, same as in Fig 3B. F) Layout of the *glyS* T-box, with the location of the probes (A, B, C and D). The lines below show the various detected RNA species. TB: Full-length T-box RNA. *: A ~125 nt degradation product that accumulates in the ΔY and Y^367AA^ strains and appear to be detectable by all four probes. #: A short (~25 nt) that is only detected by probe glyS-B. Fragment Y is readily detected in strains J1^AGA^, using probe glyS-C (Fig 5H), but only overlap partially with probe glyS-D, and therefore gives a poor signal.
(PDF)

S8 Fig *rpsB* mRNA is endonucleolytically cleaved via two separate pathways. Northern blots of the *rpsB* transcript, using a 8% acryl-amide gel to obtain high resolution of short RNA fragments. Panels A to D show the results with probes A to D, respectively. Panel C and D are identical to Fig 4G and 4H. For probe C note the ~75 nt RNA that accumulate in the J1^AGA^ mutant. (E) Overview of the *rpsB-tsf* locus, with the various predicted transcription start sites (TSSs) indicated. The four probes used for Northern blotting are shown in grey, and the location of the fragment detected with probe C in the J1^AGA^ mutant is highlighted by a thick black arrow. Thin arrows indicate open reading frames, including the short unannotated ORF *X*. RNase Y cleavage sites are marked in bold, and the question mark indicates the frequent read-through of the putative transcriptional terminator located inside ORF *X*. (F) Loading control using a probe against 5S rRNA, same as Fig 3B. (G) The two proposed pathways for degradation of *rpsB* RNA.
(PDF)

S9 Fig Northern blot of *rplS*. A) Northern blot with probe rplS-A. B) Northern blot with probe rplS-B. C) Northern blot with probe rplS-C. D) 5S rRNA loading control, same as in Fig 3B. E) Layout of the *rplS* gene, with the location of probes A, B and C indicated.
(PDF)

S1 Table Steady-state and half-life changes of ORFs and ncRNAs in the ΔY mutant. (XLS)

S2 Table List of the 99 identified RNase Y cleavage sites, and additional information about the EMOTE data. (XLS)

S3 Table Full EMOTE data for unambiguously mapping reads. Seqnames: Accession number for the reference genome used. 5prime pos: Position on the genome where the first (5’-end) base of the RNA maps. Strand: Strand to which the RNA correspond. Nuc5p: The first (5’-end) base of the RNA. N: Number of reads that correspond to that specific 5’-end. Q: Quantification value, i.e. number of unique Quantification Sequences, for that specific 5’-end (see also Redder, 2015 for a detailed explanation).
(ZIP)

S4 Table Full EMOTE data for ambiguously mapping reads. Seqnames: Accession number for the reference genome used. 5prime pos: Position on the genome where the first (5’-end) base of the RNA maps. Strand: Strand to which the RNA correspond. Nuc5p: The first (5’-end) base of the RNA. N: Number of reads that correspond to that specific 5’-end. Q: Quantification value, i.e. number of unique Quantification Sequences, for that specific 5’-end (see also Redder, 2015 for a detailed explanation).
(ZIP)
