# Correction: Decay-Initiating Endoribonucleolytic Cleavage by RNase Y Is Kept under Tight Control via Sequence Preference and Sub-cellular Localisation

**DOI:** 10.1371/journal.pgen.1006320

**Published:** 2016-09-14

**Authors:** Vanessa Khemici, Julien Prados, Patrick Linder, Peter Redder

Fig 5 is incorrect. The authors have provided the correct Fig 5 here.

**Fig 5:**
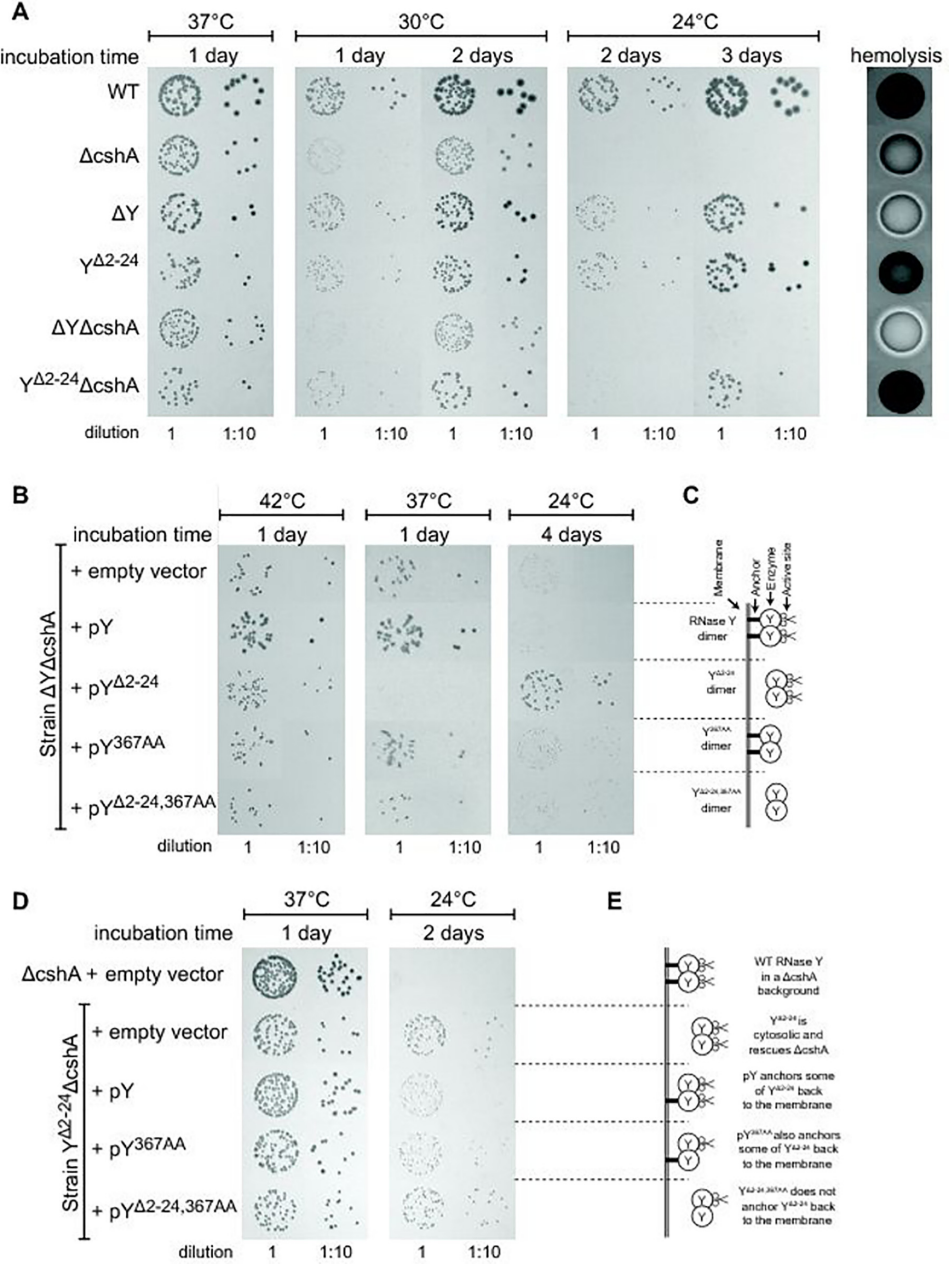
Removal of the membrane anchor enables RNase Y to suppress the phenotypes of a ΔcshA mutant. (A) In the left panel, over-night cultures of single mutants ΔcshA, ΔY and Y^Δ2–24^ and double mutants ΔYΔcshA and Y^Δ2–24^ΔcshA were diluted, spotted on agar-plates, and incubated at the indicated temperatures and times. In the right panel, over-night cultures were spotted on horse-blood-agar. (B) Transformants of strain ΔYΔcshA with plasmids expressing variants of RNase Y were selected at 42°C, then restreaked and grown over night at 42°C. Finally the cultures were diluted and spotted at the indicated temperatures. ΔYΔcshA with pY^Δ2–24^ grows significantly better than the other strains at 24°C. (C) Cartoon showing the four versions of RNase Y expressed from the plasmids; wild-type RNase Y (pY), anchorless RNase Y (pY^Δ2–24^), RNase Y active site mutant (pY^367AA^), and anchorless RNase Y active site mutant (pY^Δ2–24,367AA^). (D) The Y^Δ2–24^ΔcshA strain was transformed with the plasmids expressing the wild-type RNase Y, Y^367AA^ or Y^Δ2–24,367AA^. Overnight cultures were diluted, spotted on agar-plates and incubated at the indicated temperatures for the indicated period of time. Both pY and pY^367AA^ inhibit growth at 24°C. (E) Cartoon showing how the wild-type RNase Y and Y^367AA^ can anchor the Y^Δ2–24^ protein back to the membrane, via dimer-formation.
