# Supplementary material for: Decay-Initiating Endoribonucleolytic Cleavage by RNase Y Is Kept under Tight Control via Sequence Preference and Sub-cellular Localisation
Source: PLoS Genet. 2015 Oct 16;11(10):e1005577. doi: 10.1371/journal.pgen.1005577 (PMC4608709; doi:10.1371/journal.pgen.1005577)
Supplement: S1 Text — (PDF) [file pgen.1005577.s001.pdf]

SUPPLEMENTAL MATERIALS AND METHODS:

Strains and plasmids:

The various mutants were constructed as described in Redder and Linder, 2012. The insert for double homologous recombination to generate strain PR01-03 was made by fusing the PCR products from primers RNaseY-Eco-F1+RNaseY-rTailfus-R1 and RNaseYrT-Nar-R1+RNaseY-rTailfus-F1 and cloning it into vector pRLYE1. For strain PR01-69 the primer sets were RNaseY-Xho-F1+RNaseY-AA-Fus-R1 and RNaseY-R-Nar-R1+RNaseY-AA-Fus-F1.

For complementation, RNase Y and mutants thereof were cloned together with the natural *cvfA* promoter, into expression vector pEB01, by sub-cloning into pCG to add a StrepFlagHis tag at the C-terminus of the proteins.

pVK17 was constructed as follows: A first PCR fragment was made by two-fragment fusion-PCR using the oligonucleotides RNaseY-salF3+OVK51 on wild-type genomic DNA for fragment 1 and OVK14+OVK16 on pCG (Giraud et al 2015) for fragment 2. Using a mixture of fragment 1 and 2 as template, the fusion-PCR was amplified with RNaseY-salF3 and OVK57. The product was digested and cloned into the pEB01 plasmid between the SalI and AscI restriction sites. pVK48 and pVK49 were made by a two-fragment fusion-PCR. For pVK48 fragment 1 was amplified using RNaseY-salF3+RNaseY-rtailfus-R1 and the second using RNaseY-rtailfus-F1+OVK57. For pVK49 one fragment was amplified using RNaseY-salF3+RNaseY-aa-fusR1 pair and the second using RNaseY-aa-fusF1+OVK57 pair. For pVK50 a 3 fragments fusion-PCR was amplified using 3 pairs RNaseY-salF3+RNaseY-rtailfus-R1, RNaseY-rtailfus-F1+RNaseY-aa-fusR1 and RNaseY-aa-fusF1+OVK57. All PCRs were performed using pVK17 as a template, digested and cloned into pEB01 between the SalI and AscI restriction sites. Plasmids have been verified by sequencing the regions between the restriction sites.

Strains:

Strain	Name	Description	Parent strain	Reference
WT	PR01	Derivative of clinical strain SA564, Δ*pyrFE* and restriction deficient	-	Redder and Linder 2012
ΔY	PR01-02	PR01 with a Δ*cvfA* mutation	PR01	Redder and Linder 2012
Y^{367AA}	PR01-69	PR01 with *cvfA*$^{H367A,\ D368A}$	PR01-02	This work
$Y^{\Delta 2\text{-}24}$	PR01-03	PR01 with *cvfA*$^{\Delta 2\text{-}24}$	PR01	This work
ΔcshA	PR01-15	PR01 with *ΔcshA::kanA*	PR01	Linder et al. 2014
ΔYΔcsh	SVK10	Replacement of *cshA* allele using	PR01-02	This work

A		pRLYE-85RL-Kan-1 vector		
$Y^{\Delta 2-24}\Delta$cshA	SVK7	Replacement of *cshA* allele using pRLYE-85RL-Kan-1 vector	PR01-03	This work
$J1^{AGA}$	PR01-27	PR01 with *rnjA*^{H74A,H76A}	PR01	Linder et al. 2014

Plasmids and vectors:

Name	Insert	Backbone	Reference
pRLYE1	none	pRLYE1	Redder and Linder 2012
pRLYE-85RL-Kan-1	Insert for generating Δ*cshA::kan* (SVK7 and SVK10)	pRLYE1	Linder et al. 2014
pRLYT-YAA-1	Insert for generating *cvfA*$^{H367A,\ D368A}$ (PR01-69)	pRLYE1	This work
pRLYE-YrT-EN-1	Insert for generating *cvfA*$^{\Delta 2-24}$ (PR01-03)	pRLYE1	This work
pEB01	none	-	Oun et al. 2013
pCG	none	-	Giraud et al. 2015
pVK17	Y(a)	pEB01	This work
pVK48	$Y^{\Delta 2-24}$(a)	pEB01	This work
pVK49	Y^{367AA}(a)	pEB01	This work
pVK50	$Y^{\Delta 2-24,367AA}$ (a)	pEB01	This work

a) Proteins are C-terminally tagged by two streptavidine-tags, a FLAG-tag and a His_6-tag.

Oligos:

Name	Sequence, 5' to 3'
For EMOTE	
Rp6	CGGCACCAACCGAGGVVVVVVVCGC (RNA, V = A, C or G)
DROAA	GGCATTCCTGCTGAACCGCTCTTCCGATCTNNNNNNNNNAA
D6A:	CTCTTTCCCTACACGACGCTCTTCCGATCTNTACACGGCACCAACCGAGG
D6B:	CTCTTTCCCTACACGACGCTCTTCCGATCTNGTATCGGCACCAACCGAGG
D6C:	CTCTTTCCCTACACGACGCTCTTCCGATCTNCGTCCGGCACCAACCGAGG
D6D:	CTCTTTCCCTACACGACGCTCTTCCGATCTNAAGTCGGCACCAACCGAGG
D6H:	CTCTTTCCCTACACGACGCTCTTCCGATCTNTCGGCGGCACCAACCGAGG
D6I:	CTCTTTCCCTACACGACGCTCTTCCGATCTNCAAGCGGCACCAACCGAGG
D6J:	CTCTTTCCCTACACGACGCTCTTCCGATCTNTTGACGGCACCAACCGAGG
D6K:	CTCTTTCCCTACACGACGCTCTTCCGATCTNGCTGCGGCACCAACCGAGG

A-PE-PCR10	AATGATACGGCGACCACCGAGATCTACACTCTTTCCCTACACGACG
B-PE-PCR20	CAAGCAGAAGACGGCATACGAGATCGGTCTCGGCATTCCTGCTGAACCGC
For plasmid and strain construction	
RNaseY-Eco-F1	TAGACGGAATTCGCGGGGTGGCAGCATTTA
RNaseYrT-Nar-R1	AACGGTGGCGCCCGTTTCGCTAATGTCTCATCT
RNaseY-rTailfus-F1	GTGTTTGTGTGCGAAATTTGTTGCTTCAAAAGCAATC
RNaseY-rTailfus-R1	CAACAAATTTCGCACACAAACACCTCCTTTTCTAGGG
RNaseY-Xho-F1	GACGCTCGAGGCGGGGTGGCAGCATTTA
RNaseY-R-Nar-R1	CCTAAGGCGCCGTCATAAAAACCTGTCATACC
RNaseY-AA-Fus-F1	CGAGCTGGACTTTTAGCTGCTGTTGGTAAAGCAAT
RNaseY-AA-Fus-R1	CCAACAGCAGCTAAAAGTCCAGCTCGTTTCGC
OVK14	TTAATGGTGATGGTGATGATGACCGCCTTTATCATCATCATCTTTATAATCCTCTC
OVK16	AAACCCGGTAGCTGGAGC
OVK51	GTGGCTCCAGCTACCGGGTTTCGCATATTCTACTGCTCTAGTCT
OVK57	ATCAGGCGCGCCTTAATGGTGATGGTGATGATGACCG
RNaseY-salF3	CCCAGGTCGACGAAACAGAAGATGCACCAAAG
Probes for Northern blotting	
valS-TB-A	TTCAAGAAGGATGTTTAATATCAAATTCACTCTTTTAAC
leuS-TB-A	CACCTCTAACTATTCTATTTACATAGGGACGACATTTGC
serS-TB-A	CCCTATTTAACAATTTAAGTTATAAAGATACACTAAACC
glyS-TB-A	GCCAATCTTATTGTTAAGCTTACACTATCCTTAACTCGC
glyS-TB-B	CGGTTCCACCCAAATTAGTGTAGTCACTCGCTTTTATTT
glyS-TB-C	ATGGGACGAGTTAATATTTTAAATTGTATATAATACAAA
glyS-TB-D	CGCCCCATGGAAAATAACAGCATAGTAAATATGCTTTAC
rpsB-A	TAAATATTGCACCCTTTACACAATCACGTTTGATATGTG
rpsB-B	CAGAAATACGTTTGATTCGTTTTGAGATTGTTTTATAGT
rpsB-C	CTAAGTCAGATTCAACAGTTGATTATTCTTCAGTTGCTT
rpsB-D	AGATTGCCAATACTCTAATTGAATATTTGTAATTTGCCT
rplS-A	TCAACCTATGTTCTTGCCTCGACAAATATATAGCAGCGG
rplS-B	TCGAATACTTGGATACGCTCACGAGTACCCTCAATGATA
rplS-C	AGTGACCTAGATGGCTTTATTGTTTGAGTCGAATTTGAC
tsf-E	GCCGCAATACGGTCTGCTTTTTTAGCTGCTTTAGCAATA
5S rRNA	TTAACTTCTGTGTTCGGCATGGGAACAGGTGTGACCTCC
Primers for RT-PCR	

SA0941-F	CTGAAATTTTAGTGTTATTTCAC
SA0941-R	AACTAGATATGTGTAGTCAGGT
TSF-F	CAAACGACGATGCTATCCG
TSF-R	TTCTTTAACTAACTCTTGGAATC
rpsO-F	GAAACTGATACTGGTTCAC
rpsO-R	CATCTTTATCTCATTGTAAC
SA1735-F	TCGTAGATACAAGTGGTAGAG
SA1735-R	GAGTTTCTGCACTCACATCACC
FER-F	GTCAAAAAAGGTTTGGTGAG
FER-R	TCCCCATCAAATGATTCGTC
SA0802-F	CTTTTTTGTGAACATCACAGG
SA0802-R	GCCGAATGTTTCACTAACGA
FUSTUF-F	CGTTCAAACACTCAAGGTC
FUSTUF-R	TATTGATTGTGATACCACG
SA1186-F	CCGAACGAAATGCGCTTAT
SA1186-R	GGTCATCTTCAAAGTACCAC
SA2095-F	CGCATCATTTTTATATTGTC
SA2095-R	GATGCACTTCAGGATTACC
SPX-F	ATGTTTTTTCTTGATCTATAACTA
SPX-R	CAACCATACGTTGTGCTTC
cvfA-F	GTATGATTTATATATAATTTCAT
cvfA-R	TTCTTCTTGAGTGAGACCG
glyS-F	CTACACTAATTTGGGTGGAAC
glyS-R	TACTTACAGGACAAACAATAC
valS-F	TGAAGGTAGCCCACAGAG
valS-R	CTTTCCAATCCCATGCCTG
SA0641-F	TACCACTACCCAAAAATCCG
SA0641-R	GAAGCGACTTTGTCAGATGC
Primers for qRT-PCR	
HU_1687F	GGTTTCGGTAACTTTGAGG
HU_1747R	CAGTTTGAGGGTTACGACC
AgrA-34F	CAAAGAGAAAACATGGTTACCATTATTAA
AgrA-135R	CTCAAGCACCTCATAAGGATTATCAG

Growth and media:

All strains were grown in Mueller-Hinton broth (Becton-Dickinson) with uracil (20 mg/l) and appropriate antibiotics where indicated, at the following concentrations: 10 mg/l chloramphenicol, 10 mg/l erythromycin, 50 mg/l kanamycin.

Growth-curves were generated by measuring the OD_{600} every 5 minutes in a Synergy H1 plate reader (Biotek), agitating for 30 seconds before each measurement. Cultures were assayed in quadruplicate in a volume of 200 µl.

Spot-assay: In the spot-assay, the size of the colonies indicates semi-quantitatively how well a strain is growing at a specific growth condition. This assay has the advantage over growth-curves

performed on liquid cultures, that spontaneous suppressor mutations are extremely unlikely to affect the analyses.
Hemolysis assay: 5 or 10 µl of over-night cultures were plate on Columbia gelose containing 5% horse-blood plate (Biomerieux), and incubated over-night at 37°C. Note that while a halo of hemolysis around the ΔY strain is readily observable after an over-night incubation at 37°C, to observe halo of hemolyses in the ΔcshA strain a further incubation for 1 or 2 days at 4°C is necessary.
For RNA isolation, cultures were harvested in mid-exponential phase at an OD_{600} of ~0.4, by mixing with a large volume of ice-cold ethanol/acetone (50% v/v), thereby immediately blocking further RNA decay as well as killing the cells. Cells suspended in ethanol/acetone were pelleted (4000g for 5 minutes), the supernatant was removed, and 1 ml of ethanol/acetone was added. Samples were then stored at -80°C until RNA isolation (see below).
For the Rifampicin experiments, a sample was taken as described above at OD_{600} of ~0.4 (time-point 0s), whereupon 400 µg/ml Rifampicin (Sigma) was added, and further samples were taken after 150s, 300s and 600s.

RNA isolation:
Cells suspended in ethanol/acetone were pelleted (4000g for 5 minutes), washed in 1 ml TE buffer, resuspended in 200 µl TE with 100 µg Lysostaphin (Sigma) and 40 U RnasinPlus (Promega), and incubated at 37°C for 10 minutes.
The lysed cells were mixed with 350 µl RLT buffer from the RNeasy Mini Kit (Qiagen), added to a QIAshredder spin column (Qiagen) and centrifuged at 17000g for 2 minutes. The flow-through was mixed with 250 µl ethanol, and the manufacturer's instructions for the RNeasy Mini Kit were followed.
For the acryl-amide Northern blot analysis, it was important to quantitatively purify even small RNA fragments, and the lysed cells were therefore in this case mixed with 600 µl Trizol Reagent (Ambion), and the manufacturer's instructions followed, to isolate the RNA.

Decay and steady-state analyses:
Quantification, normalization, and decay half-lives were computed for each assay following the procedure described in (Giraud et al. 2015). Briefly, reads are mapped to the *S. aureus* N315 genome with the software BWA (Li and Durbin, 2009). A script in the R programming language, counted the number of them that overlaps a gene, and discarded those that align to multiple positions on the genome or on the inappropriate strand. A normalization step further adjusted genes

counts according to the level of expression of the housekeeping gene HU, which has a very long half-life. Half-lives were then obtained by fitting a weighted linear model on log2-transformed expression values of each gene.

Quality of the half-life estimates were asserted by checking for a minimum read count of 100, on average over the four time points, and a minimum value of 20 on the fitted error of the linear models. Additionally, duplicated experiments of the WT assay and the ΔY assay were compared pairwise in a smallest difference model to determine the most conservative fold change in half-life and in steady state level for each gene.

EMOTE assay:

Ribosomal depletion of ~4 µg RNA was performed with the MICROBExpress kit (Ambion) or RiboZero (Epicentre), and the EMOTE protocol described previously (Redder, 2015) was followed up to and including the reverse transcription step, whereupon the following modification was made to the second-strand PCR reaction in order to immediately add the full-length adaptors required for Illumina sequencing:

10 µl PCR-purified RT-reaction, 27 µl H2O, 10 µl Q5 buffer, 1.5 µl dNTP (2.5mM each), 1.5 µl 10 µM Primer B-PE-PCR20, 0.5 µl Q5 HotStart enzyme (New England Biolabs), and 1.5 µl 100 nM of either primer D6A, D6B, D6C, etc.

The 50 µl PCR reaction with low D6x primer concentration was started. At the end of cycle 5, the PCR machine was paused, and 1.5 µl 10 µM A-PE-PCR10 was added, all tubes replaced in the PCR machine and the program continued to 31 cycles.

Program: 2 min @98°C, 31 cycles of (10s @98°C, 20s @50°C, 2 min @ 72°C), 5 mins @72°C, and then 4°C.

An 0.8% agarose gel was loaded with 10 µl of each reaction to visually verify that the yields are similar, and the PCR reactions were then pooled and purified/concentrated using the Wizard® SV Gel and PCR Clean-Up System (Promega), and size-selected to 300-1000 bp as previously described (Redder, 2015).

After sequencing a 50 nt single-run on a Illumina HiSeq2500, the resulting FastQ-file was treated with the EMOTEconv pipeline (Yasrebi and Redder, in press) and Bowtie 1.0.0 (Langmead et al. 2009) to obtain more than 600000 reads that map unambigously (see table below).

Overview of EMOTE reads:

RNA sample	Oligo	EMOTE barcode	Number of unambigously mapped reads

WT, replicate 1	D6A	TACA	1967922
ΔY, replicate 1	D6B	GTAT	1112960
Y^{367AA}, replicate 1	D6C	CGTC	919415
$Y^{\Delta 2\text{-}24}$, replicate 1	D6D	AAGT	2202132
WT, replicate 2	D6H	TCGG	616020
ΔY, replicate 2	D6I	CAAG	1079745
Y^{367AA}, replicate 2	D6J	TTGA	717535
$Y^{\Delta 2\text{-}24}$, replicate 2	D6K	GCTG	747112

Apart from acting as an anchor for amplification and sequencing, the Rp6 oligo also contains a stretch of 7 random nucleotides, the so-called Quantification Sequence, which are sequenced as part of the Illumina read. If two Illumina reads map to the exact same genomic position, then it is not immediately possible to determine whether they originate from the same ligation event, however, if their Quantification Sequences are different, then it is certain that the two reads came from different Rp6 molecules, and therefore from two different ligation events. This again means that in the original total cellular RNA preparation, there were two different RNA molecules with identical 5'-ends.

For each potential position, the following very stringent criteria were used to ensure a high level of confidence for the identified RNase Y cleavage sites:

1) Only unambiguously mapped reads are considered – This avoids counting reads twice, and at the same time eliminates ribosomal RNA from analyses.

2) For each sample, the Quantification Sequence Counts of 5'-ends detected by EMOTE are normalized by dividing with the total number of unambiguously mapping reads (more than 600000 for all samples) from the sample and then multiplied by 1000000 – This allows direct comparison between EMOTE data from different RNA samples and sequencing runs.

3) This Normalised EMOTE Quantification Count (NEQC) of the WT strain is required to be higher than 10 – Thus in each biological replicate, the same 5'-end is detected independently at least 10 times per million reads.

4) The NEQC of both the ΔY and Y^{367AA} strains are required to be 5 times less than the quantification of the WT – This selects for 5'-ends that are generated by RNase Y.

5) Constraints 3) and 4) are required for both duplicates of the EMOTE assays.

6) The number of reads from the RNA-seq of the ΔY strain that maps to a window of 300 nt surrounding the position must not be less than 2 fold lower than the number of reads in the RNA-seq data from the WT strain – This is to avoid false positives when an RNA is not expressed in the ΔY strain, which would otherwise automatically qualify all 5'-ends within this RNA with respect to constraint 4).

7) The absolute number of ΔY RNA-seq reads mapping to the 300 nt window must be larger than 200 in both biological replicates – This is to ensure that the data in 6) is within the measurable range.
8) If two or more immediately adjacent positions conform to the above criteria, then the site with the highest number of independently detected 5'-ends in the WT EMOTE data will be taken, and neighbouring positions discarded – This is to avoid bias in the following analyses, in the event that RNase Y cleaves slightly imprecisely.

Northern blotting:
For large RNA molecules: A 1.5% MOPS agarose gel with formamide was loaded with 4 µg total RNA from each sample and the RiboRuler High Range RNA Ladder (Fermentas). After electrophoresis, the RNA was capillary-blotted onto a Hybond-N nitrocellulose membrane (Amersham). Crosslinking was done in a UV Stratalinker 2400 (Stratagene). The marker, the 16S rRNA and the 23S rRNA were visualised using Methylene blue.
For small RNA molecules: A mini-gel (Bio-Rad) with 8% acryl amide and 8M urea was loaded with 5 µg total RNA from each sample and the RiboRuler Low Range RNA Ladder (Fermentas). The RNA was migrated for 2 hours at 90V and then transferred to a Hybond-N nitrocellulose membrane (Amersham). Crosslinking was done in a UV Stratalinker 2400 (Stratagene), and the marker was visualised using Methylene blue. DNA oligo probes were 5' labelled with ^{32}P and the membrane was hybridised over night at 37°C in ExpressHyb hybridization solution (Clontech, Mountain View, CA, USA), washed, and then the signal was detected using a Typhoon FLA 7000 phosphorimager (General Electric). The membrane was stripped between each re-probing by washing for 2 hours at 75°C with 0.2% SDS and 10 mM Tris pH 7.5. Loading control was done with a 5S rRNA probe.

RT-PCR:
Total RNA was purified from WT cells, using the ReliaPrep RNA Tissue Miniprep System, including the on-column DNase treatment step (Promega).
20 µg of the RNA was then treated a second time with 25 units of RQ1 RNase-free DNase (Promega), while eliminating RNase A activity with RNasinPlus (Promega).
Enzymes were removed with two phenol-chloroform extractions and Phase Lock Gel (www.5prime.com), whereupon the RNA was ethanol-precipitated and resuspended in 30 µl 0.1xTE, yielding ~700 ng/µl of RNA.
3.5 µg RNA was mixed with 250 pmol random hexamer primers, heated to 75°C for 5 minutes and allowed to cool slowly to room temperature. Then Protoscript II Reverse Transcriptase Reaction Buffer, 10 mM DTT, 400 U Protoscript II Reverse Transcriptase (New England Biolabs), 10 nmol

dNTPs and 20 U RNasinPlus was added, and the 20 µl reaction was incubated for 10 minutes at room temperature, and then shifted to 42°C for 60 minutes, whereupon the enzyme was inactivated by 30 minutes at 65°C.

PCR reaction: 28 Green GoTaq PCR reactions (25 µl) were prepared according to protocol (Promega), 2 reactions with RT-PCR primer pair (see oligonucleotide table), using either cDNA generated from 35 ng RNA in the reverse transcription described above or 140 ng RNA without reverse transcription (negative control). Annealing temperature was 50°C and elongation for 2 minutes, for 30 cycles. The PCR products were evaluated by electrophoresis in a 1.5% agarose gel.

qRT-PCR:

RNA was isolated and DNA was removed as described above for RT-PCR. 2.5 ng RNA was used in each qRT-PCR reaction with the GoTaq 1-Step RT-qPCR System (Promega) in a CFX96 C1000 Thermal Cycler (BioRad). Two biological replicates were examined, each in technical triplicates, and the signal from the *agrA* primer-pair was normalised to that from the *HU* primer-pair, as described by Oun et al. (2013).

References:

Giraud C, Hausmann S, Lemeille S, Prados J, Redder P, Linder P. The C-terminal region of the RNA helicase CshA is required for the interaction with the degradosome and turnover of bulk RNA in the opportunistic pathogen Staphylococcus aureus. RNA Biol. 2015;12(6):658-74.

Langmead B, Trapnell C, Pop M, Salzberg SL. Ultrafast and memory-efficient alignment of short DNA sequences to the human genome. Genome Biol. 2009;10(3):R25.

Li H, Durbin R (2009) Fast and accurate short read alignment with Burrows-Wheeler transform. Bioinformatics 25: 1754-1760

Oun S, Redder P, Didier J-P, François P, Corvaglia A-R, Buttazoni E, Giraud C, Girard M, Schrenzel J, Linder P. The CshA DEAD-box RNA helicase is important for quorum sensing control in Staphylococcus aureus. RNA Biol. 2013 Jan;10(1):157-65. doi: 10.4161/rna.22899.

Redder, P. and Linder. P. New Range of Vectors with a Stringent 5-Fluoroorotic Acid-Based Counterselection System for Generating Mutants by Allelic Replacement in Staphylococcus aureus. Appl Environ Microbiol. 2012a Jun;78(11):3846-54.

Yasrebi H, Redder P. EMOTE-conv: A computational pipeline to convert Exact Mapping Of Transcriptome Ends (EMOTE) data to the lists of quantified genomic positions correlated to related genomic information. J Appl Bioinform Comput Biol (in press).
