# Supplementary figures and images for: Decay-Initiating Endoribonucleolytic Cleavage by RNase Y Is Kept under Tight Control via Sequence Preference and Sub-cellular Localisation
Source: PLoS Genet. 2015 Oct 16;11(10):e1005577. doi: 10.1371/journal.pgen.1005577 (PMC4608709; doi:10.1371/journal.pgen.1005577)

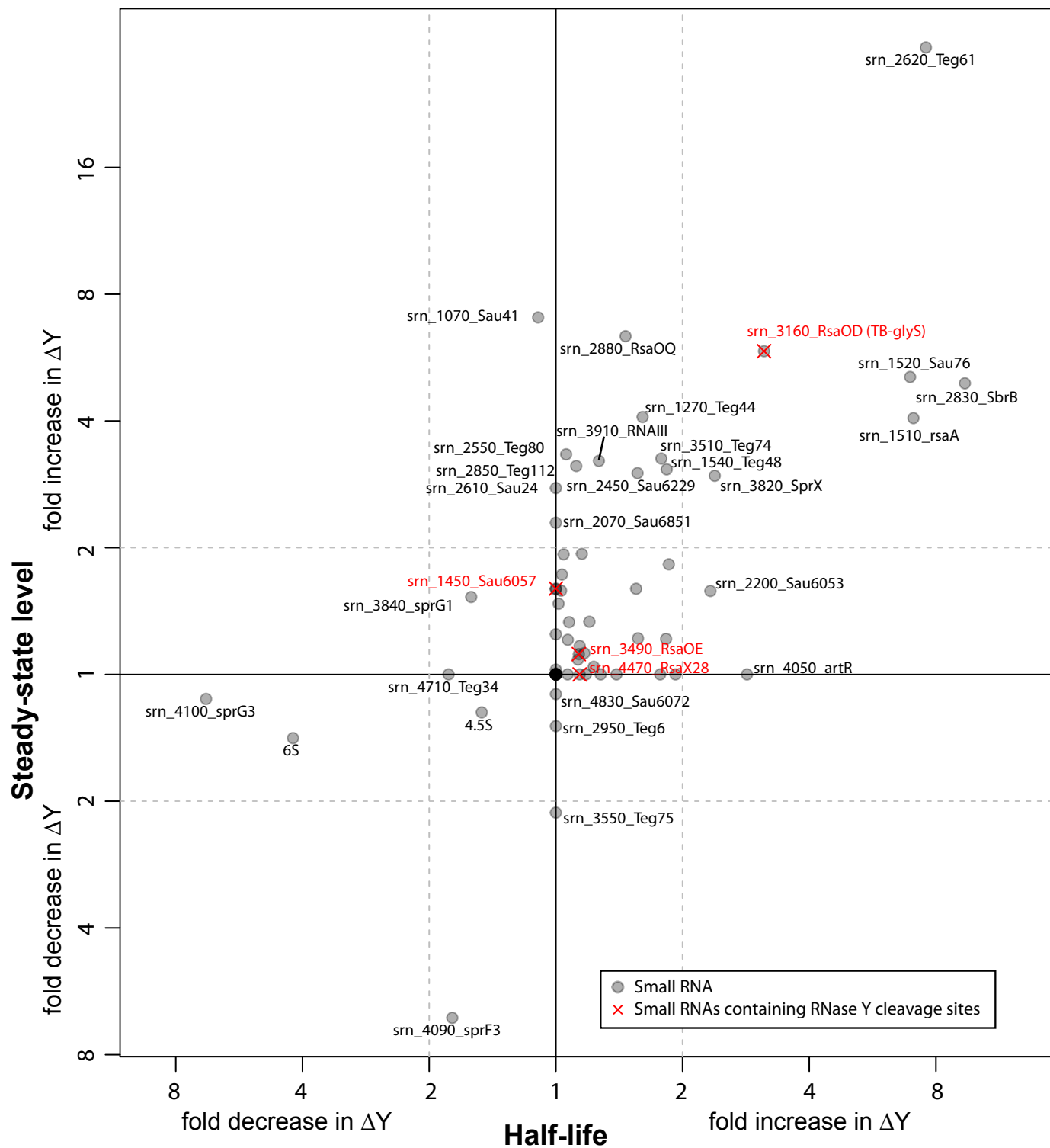

Supplement: S1 Fig — The change between WT and ΔY in the half-life of each ncRNA was estimated using a smallest-difference model, and the same was done for the steady-state levels of the ncRNAs. The fold-change in half-life is plotted on the x-axis and the fold-change in steady-state level on the y-axis. Each grey dot represents an ncRNA transcript where sufficient data was available (67 in all). Red X's show which ncRNAs contain an identified RNase Y cleavage. The list of ncRNAs was based on the database from Sassi et al. (2015). (PDF) [file pgen.1005577.s002.pdf]

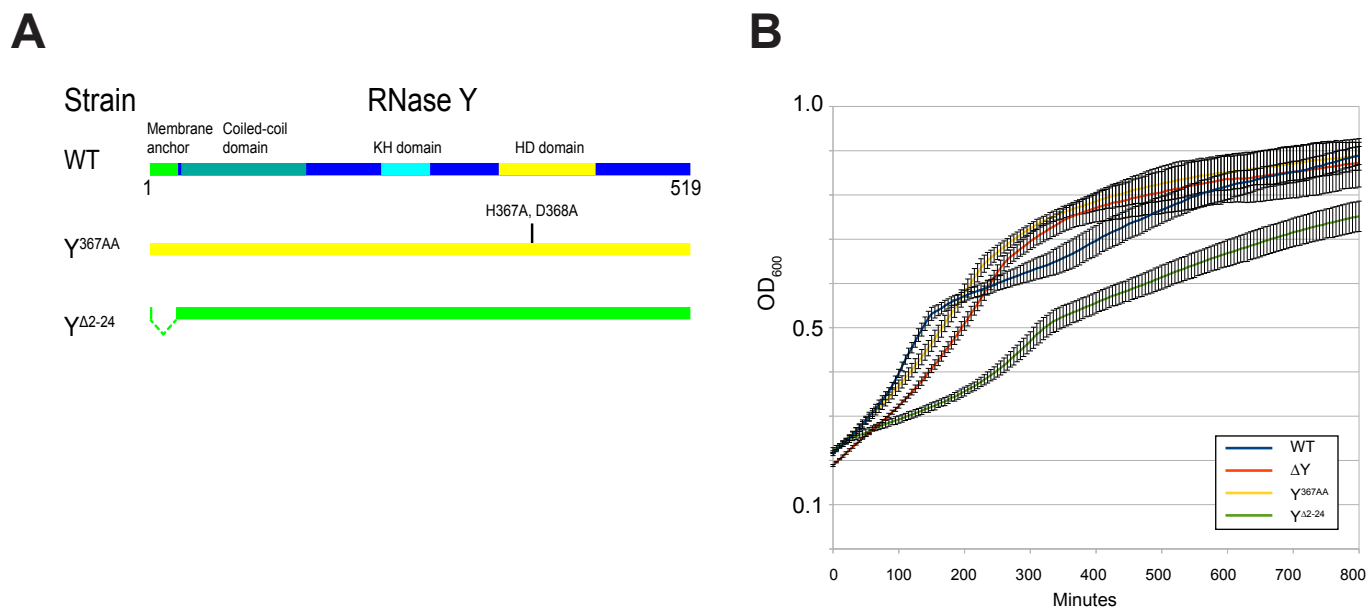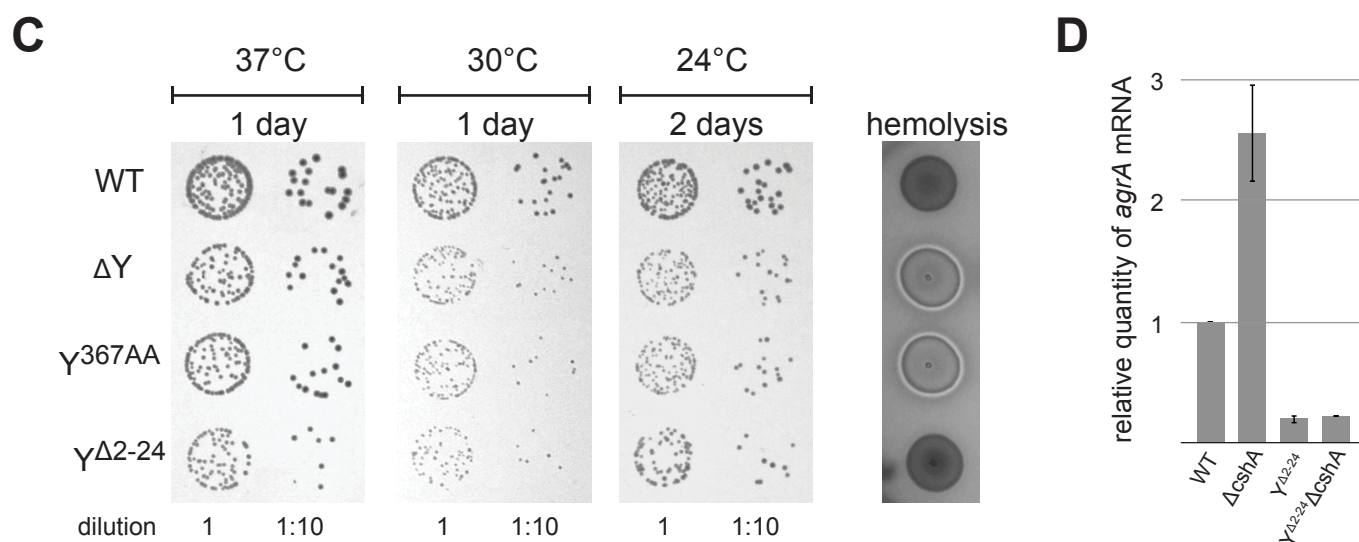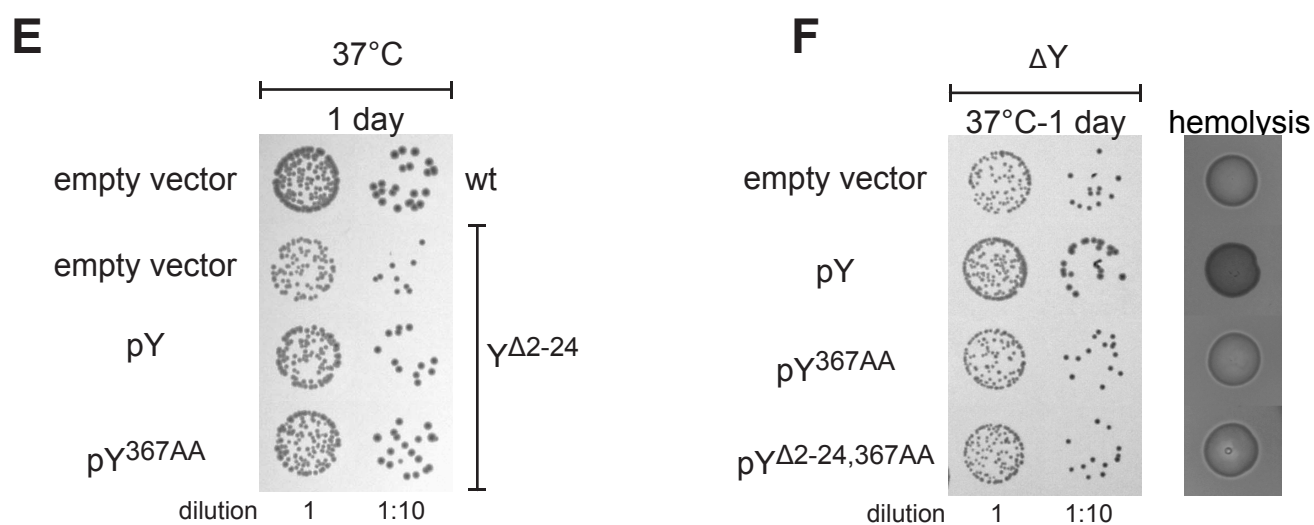

Supplement: S3 Fig — A) Overview of the RNase Y protein. The functional domains are according to Kaito et al. 2005 and Lehnik-Habrink et al. 2011: Membrane anchor, KH: RNA-binding domain, HD: Catalytic domain with the key HD motif, His367 and Asp368, that are mutated to alanines in the Y367AA mutant. B) Growth curves of WT and RNase Y mutant strains. Growth of strains was monitored continuously, in quadruplicate, in a plate-reader at 37°C. Error-bars indicate standard deviation. The cultures were started from exponentially growing cultures, however a parallel experiment started from stationary cultures gave essentially identical results. C) Growth of RNase Y variants on agar-plate. Left panel: over-night cultures grown at 37°C, were diluted and spotted on MH-agar plate at the indicated temperature for the indicated time. On agar-plate, the ΔY strain grows slightly slower than the WT strain at 37°C. The difference in growth between WT and ΔY is more pronounced at low temperature. Both ΔY and Y367AA grew equally whatever the growth condition indicating that the main effect observed with ΔY is due to its enzymatic activity as opposed to an indirect role through the binding of protein-partners. The YΔ2–24 mutant as observed in liquid culture (B) grow markedly slower at 37°C than WT and ΔY strain. Our RNA-seq analyses (Fig 1) showed that the quorum-sensing agr mRNA both accumulate and is stabilised in absence of ΔY, leading to increase production of hemolysin, which are controlled by the agr system as shown in the right panel where the strains were plated on horse-blood agar. Both ΔY and Y367AA spot have a similar halo of hemolysis, in contrast the YΔ2–24 does not induce the production of hemolysin, indicating that the YΔ2–24, still targets the agr messenger. D) qRT-PCR quantification of agrA RNA in mutants, relative to WT levels. All cultures were harvested at OD600 = 0.4, and the relative levels were normalised to mRNA of the HU housekeeping gene. Error-bars indicate the SEM. E) Growth of [file pgen.1005577.s004.pdf]

A

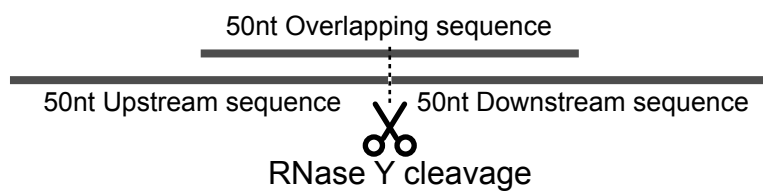

B

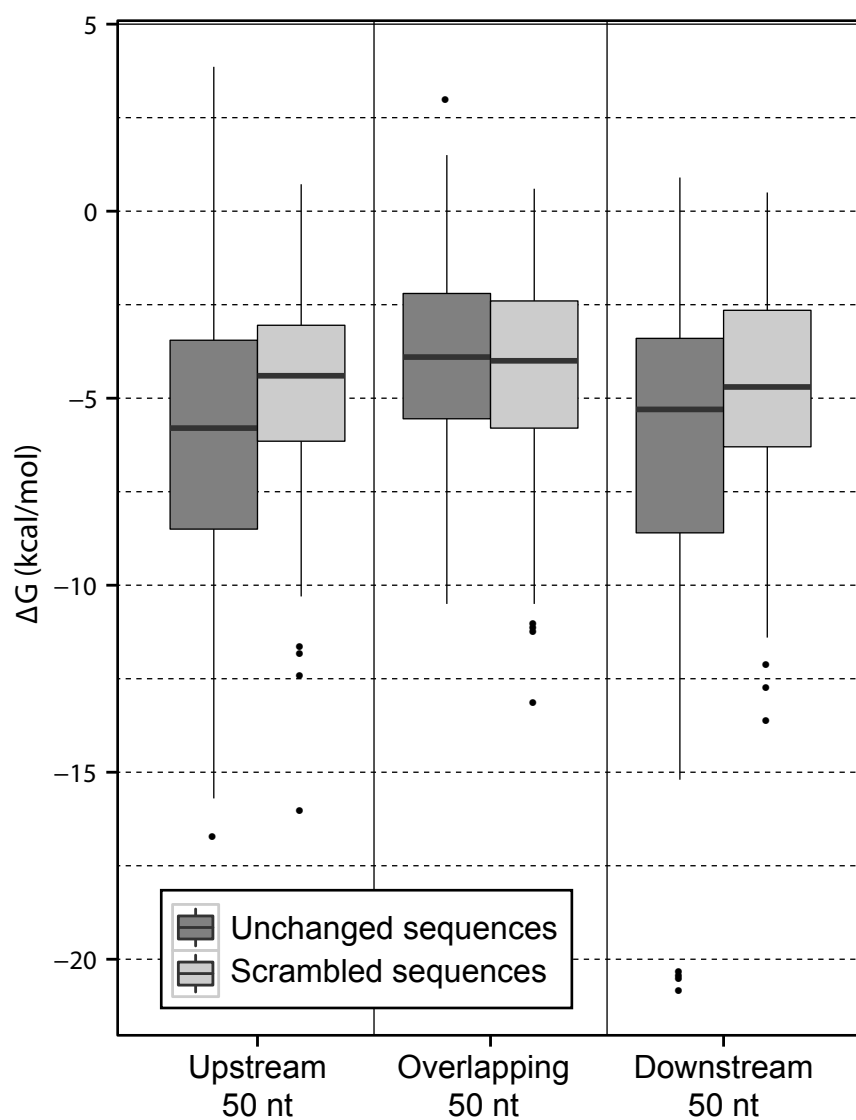

Supplement: S6 Fig — A) The sub-sequences used for calculating predicted secondary structures near the 99 RNase Y cleavage sites. 50 nt upstream, 50 nt overlapping and 50 nt downstream of the sites. B) Box-plot showing the potential free energy of folding in the three sub-regions (dark grey). One sub-region of 50 nt upstream of the site (Upstream), one region of 50 nt straddling across the site (Overlapping), and one region of 50 nt downstream of the cleavage site (Downstream). As control, the sequences for each sub-region were scrambled, and the free energy was calculated (light grey). The thick black line shows the median, the grey boxes the 25% and 75% quartiles and the error-bars are an estimate of the 95% confidence interval. Dots show the position of individual outliers. (PDF) [file pgen.1005577.s007.pdf]

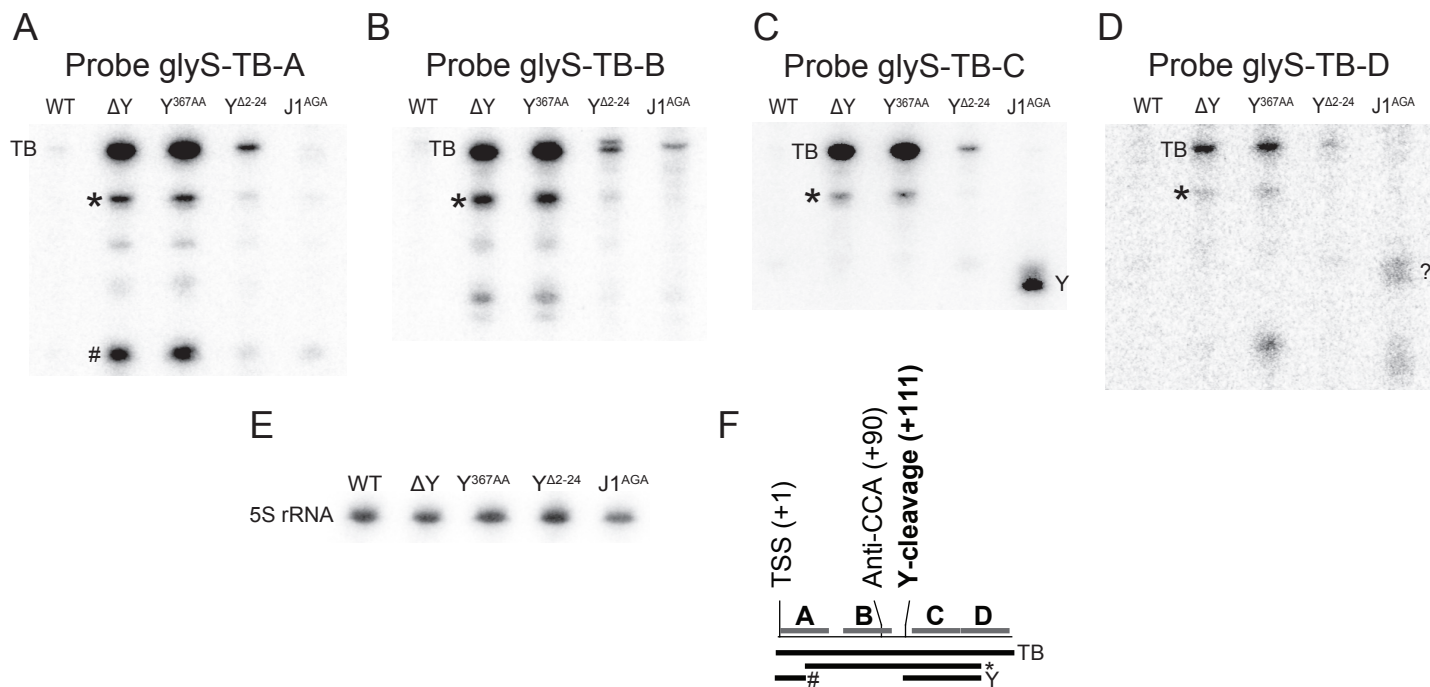

Supplement: S7 Fig — A) Northern blot with probe glyS-TB-A. B) Northern blot with probe glyS-TB-B. C) Northern blot with probe glyS-TB-C (Same exposure is shown in Fig 5H). D) Northern blot with probe glyS-TB-D. E) 5S rRNA loading control, same as in Fig 3B. F) Layout of the glyS T-box, with the location of the probes (A, B, C and D). The lines below show the various detected RNA species. TB: Full-length T-box RNA. *: A ~125 nt degradation product that accumulates in the ΔY and Y367AA strains and appear to be detectable by all four probes. #: A short (~25 nt) that is only detected by probe glyS-B. Fragment Y is readily detected in strains J1AGA, using probe glyS-C (Fig 5H), but only overlap partially with probe glyS-D, and therefore gives a poor signal. (PDF) [file pgen.1005577.s008.pdf]

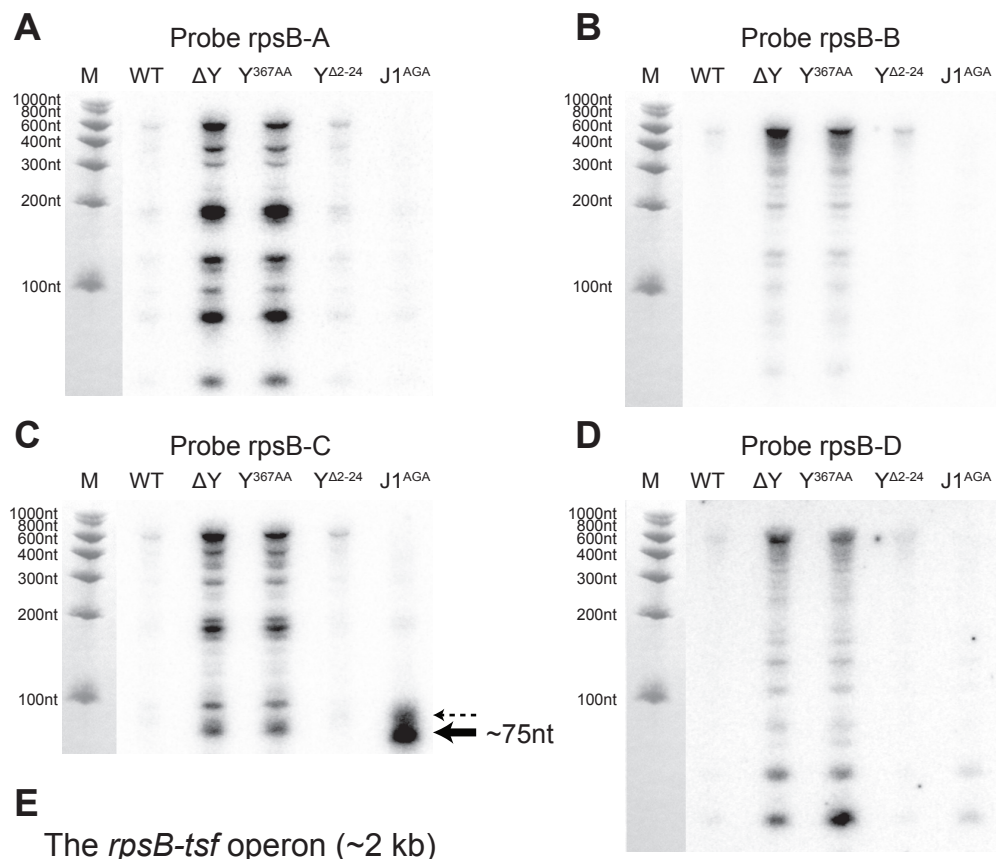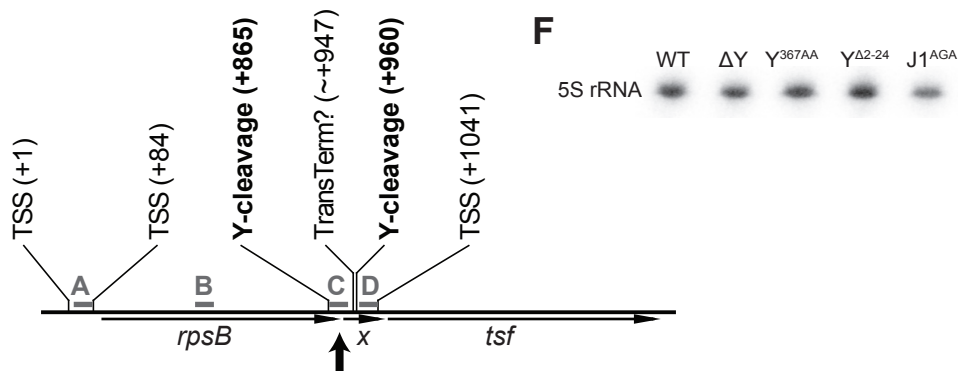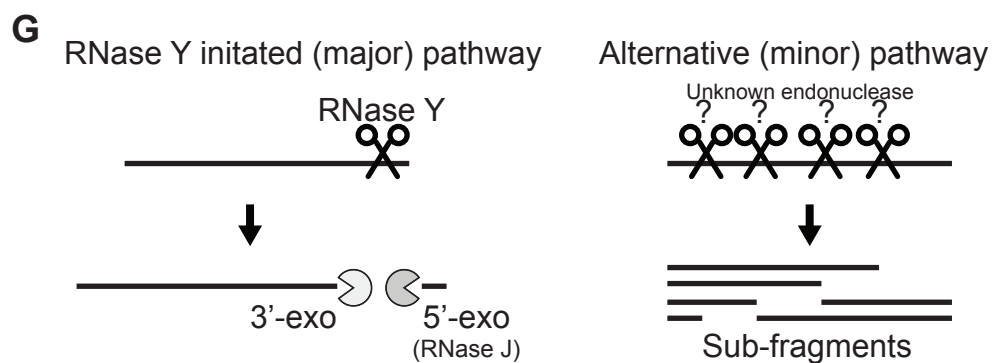

Supplement: S8 Fig — Northern blots of the rpsB transcript, using a 8% acryl-amide gel to obtain high resolution of short RNA fragments. Panels A to D show the results with probes A to D, respectively. Panel C and D are identical to Fig 4G and 4H. For probe C note the ~75 nt RNA that accumulate in the J1AGA mutant. (E) Overview of the rpsB-tsf locus, with the various predicted transcription start sites (TSSs) indicated. The four probes used for Northern blotting are shown in grey, and the location of the fragment detected with probe C in the J1AGA mutant is highlighted by a thick black arrow. Thin arrows indicate open reading frames, including the short unannotated ORF X. RNase Y cleavage sites are marked in bold, and the question mark indicates the frequent read-through of the putative transcriptional terminator located inside ORF X. (F) Loading control using a probe against 5S rRNA, same as Fig 3B. (G) The two proposed pathways for degradation of rpsB RNA. (PDF) [file pgen.1005577.s009.pdf]

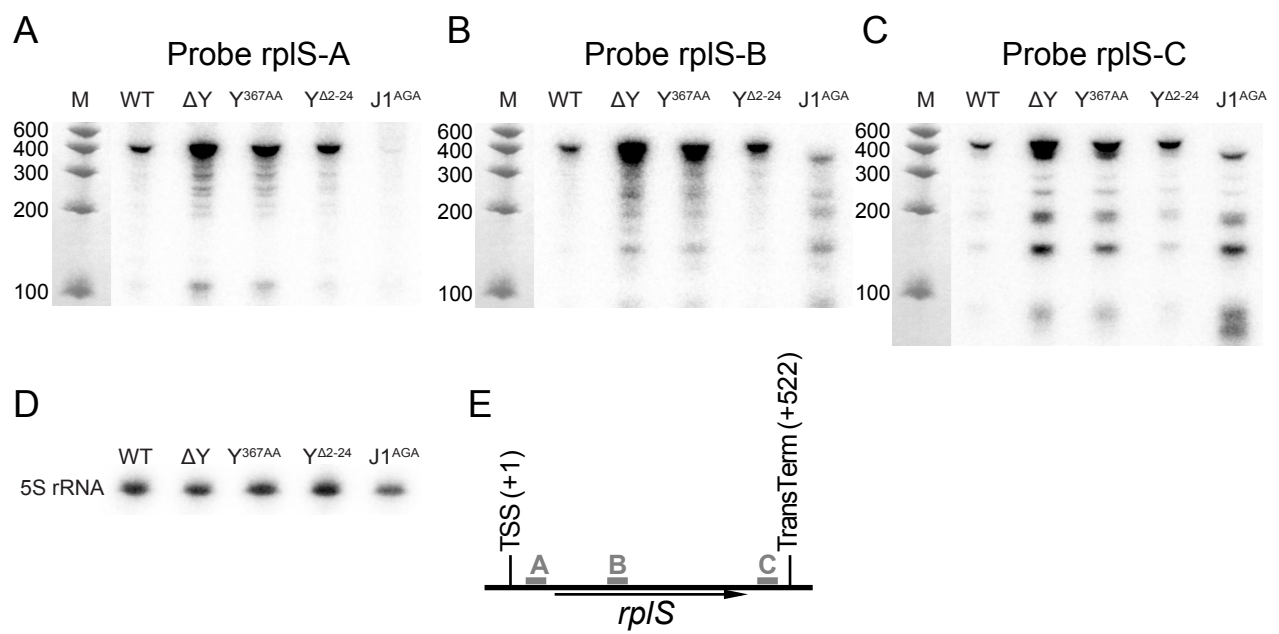

Supplement: S9 Fig — A) Northern blot with probe rplS-A. B) Northern blot with probe rplS-B. C) Northern blot with probe rplS-C. D) 5S rRNA loading control, same as in Fig 3B. E) Layout of the rplS gene, with the location of probes A, B and C indicated. (PDF) [file pgen.1005577.s010.pdf]
