# Supplementary material for: Decay-Initiating Endoribonucleolytic Cleavage by RNase Y Is Kept under Tight Control via Sequence Preference and Sub-cellular Localisation
Source: PLoS Genet. 2015 Oct 16;11(10):e1005577. doi: 10.1371/journal.pgen.1005577 (PMC4608709; doi:10.1371/journal.pgen.1005577)

Figure S4, RNase Y cleavage sites and ORFs are on the same RNA molecules.

A

Connection shown by RNAseq			
Cleavage position	ORF name	Distance to ORF	Spanning Reads #
832634	*gapR*	1	14603
835032	*pgk*	7	4803
577452	*rplA*	12	16938
2256154	*SA1985*	13	469
2256120	*asp23*	15	417
1338524	*SA1176*	17	188
1615364	*dnaK*	18	821
1708877	*tig*	22	900
504314	*dnaX*	22	261
1104307	*rpmF*	24	8082
2256134	*asp23*	29	538
1608932	*rpsU*	35	118
1773826	*tyrS*	36	81
587364	*SA0502*	41	440
587370	*SA0502*	47	323

B

Connection shown by RT-PCR		
Cleavage position	ORF(s) spanned by RT-PCR	Distance to ORF(s)
2356447	*SA2095*	37
970793	*spxA*	42
1281913	*cvfA*	54
1266149	*rpsO*	74
1246042	*rpsB, tsf*	75, 106
1353255	*SA1186*	111
905860	*SA0802*	113
1600261	*glyS*	115
590659	*fus, tuf*	85, 131
590703	*fus, tuf*	133, 87
590706	*fus, tuf*	136, 84
590651	*fus, tuf*	77, 139
736004	*SA0641*	144
1987188	*SA1734, SA1735*	0, 168
1522665	*fer*	172
1696754	*valS*	181
1245947	*rpsB, tsf*	0, 201
1987125	*SA1734, SA1735*	0, 231
1987122	*SA1734, SA1735*	0, 234
590542	*fus, tuf*	0, 248
1068776	*SA0941, rnjA*	129, 347
1068788	*SA0941, rnjA*	141, 359
1068791	*SA0941, rnjA*	144, 362

C

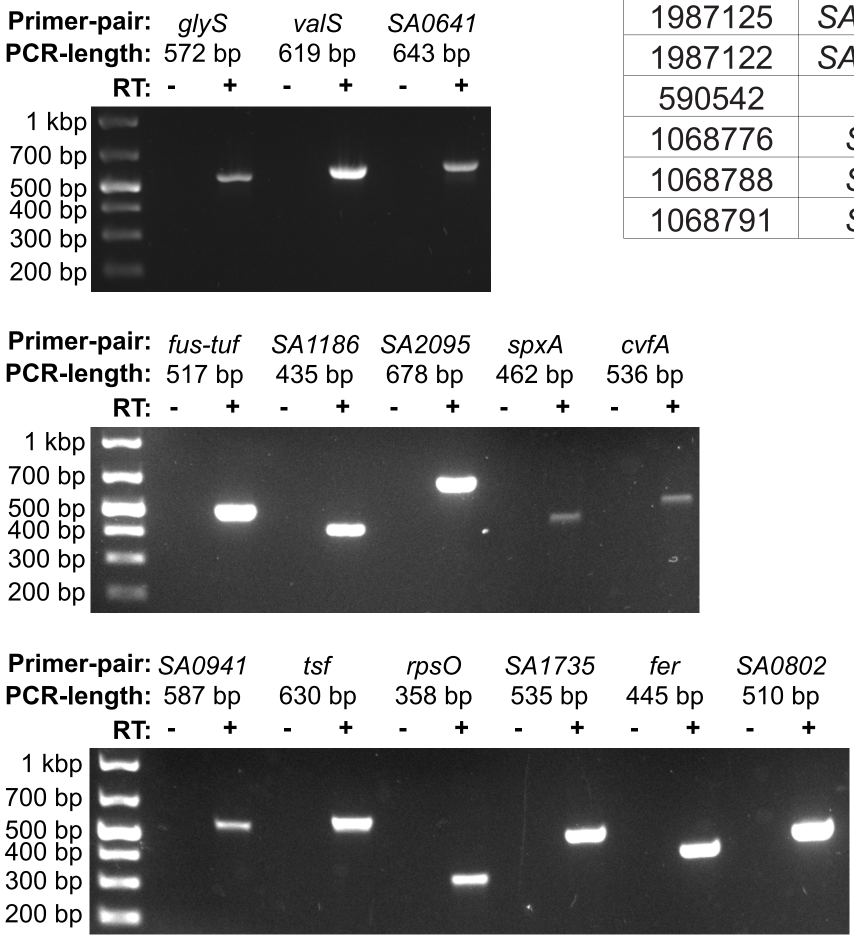

Supplement: S4 Fig — (A) List of RNase Y cleavage sites that are close enough to an ORF to prove their connection via analyses of the RNA-seq data to find 50 nt Illumina reads that span the gap (number of spanning reads shown in the last column). (B) List of RNase Y cleavage sites that fall near ORFs, and where RT-PCR was performed to demonstrate that the cleavage site and the ORF are on the same RNA molecule. Sometimes two ORFs could be connected (two ORF-names in second column), and the distance to each ORF is shown in the final column. (C) Agarose gels showing the RT-PCR product used to define (B), with each reaction performed either without or with the reverse transcriptase step. Primer sequences can be found in the oligonucleotide table. (PDF) [file pgen.1005577.s005.pdf]
