# Supplementary material for: Decay-Initiating Endoribonucleolytic Cleavage by RNase Y Is Kept under Tight Control via Sequence Preference and Sub-cellular Localisation
Source: PLoS Genet. 2015 Oct 16;11(10):e1005577. doi: 10.1371/journal.pgen.1005577 (PMC4608709; doi:10.1371/journal.pgen.1005577)
Supplement: S5 Fig — 300 nucleotides upstream and 300 nucleotides downstream of the 99 identified RNase Y cleavage sites were examined for stability in the RNAseq data, and plotted in a scatter-plot. The majority exhibits no significant stability change, however around 20 sites cleavage events clearly influence the stability of upstream and/or downstream RNA. Class 1) If both upstream and downstream fragments were stabilised by the lack of cleavage (i.e. in the ΔY mutant), then the RNase Y cleavage is presumably an initiating event for decay of the entire RNA. Class 2) If neither the stability of the upstream, nor the stability of the downstream fragment are affected by the cleavage, then the cleavage probably represents a maturation event, but may still be regulatory if the cleavage site is situated inside an ORF. Class 3) and Class 4) If either the upstream or the downstream fragment is stabilised, but the other fragment is not, then the RNase Y cleavage can serve to express co-transcribed genes differentially, by allowing the decay of either the upstream or downstream genes, thus combining maturation and decay-initiation in a single cleavage event. Thus there are multiple outcomes of RNase Y endoribonucleolytic activity. (PDF) [file pgen.1005577.s006.pdf]

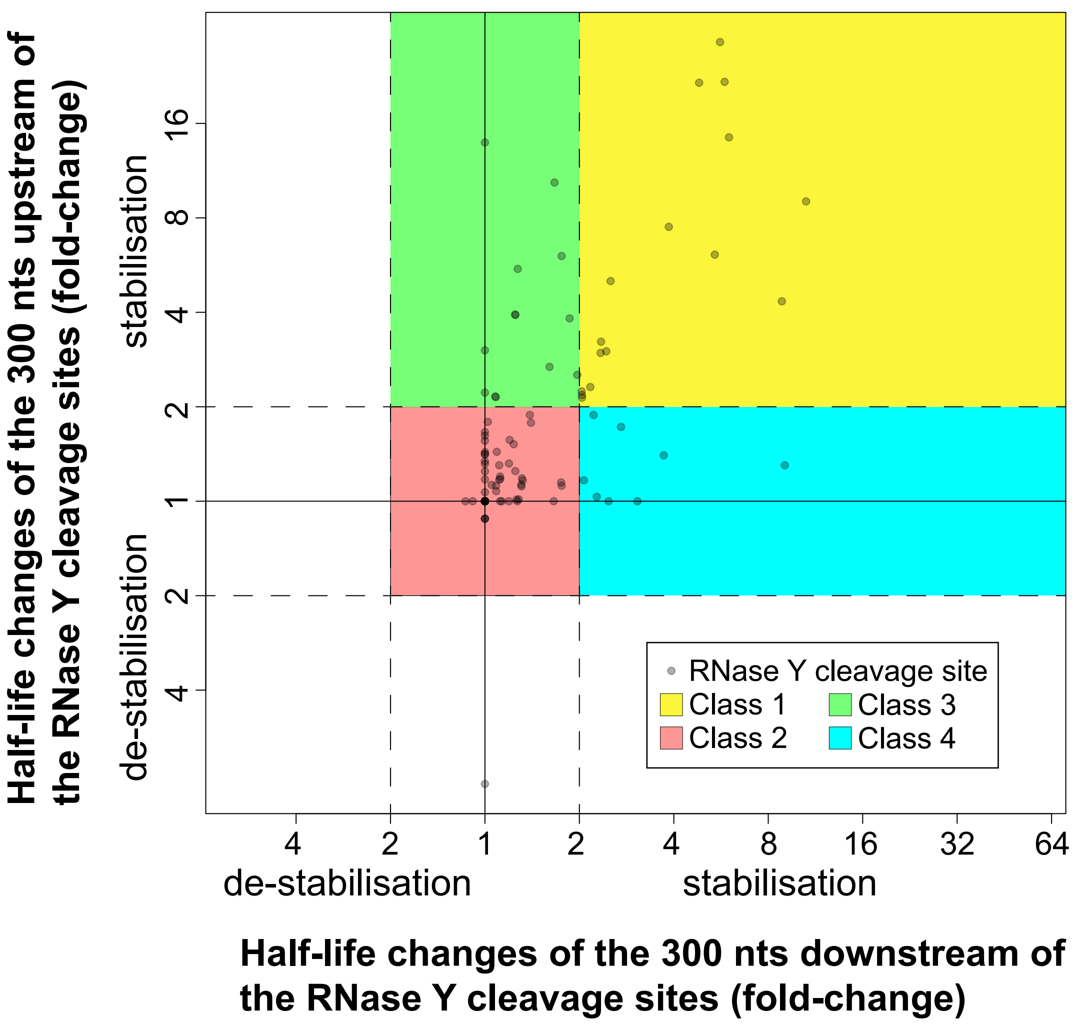

Half-life changes of the 300 nts upstream of the RNase Y cleavage sites (fold-change)
stabilisation
de-stabilisation
16
8
4
2
1
2
4
RNase Y cleavage site
Class 1
Class 2
Class 3
Class 4
4
2
1
2
4
8
16
32
64
de-stabilisation
stabilisation
Half-life changes of the 300 nts downstream of the RNase Y cleavage sites (fold-change)
