# Supplementary material for: Decay-Initiating Endoribonucleolytic Cleavage by RNase Y Is Kept under Tight Control via Sequence Preference and Sub-cellular Localisation
Source: PLoS Genet. 2015 Oct 16;11(10):e1005577. doi: 10.1371/journal.pgen.1005577 (PMC4608709; doi:10.1371/journal.pgen.1005577)
Supplement: S2 Fig — RNA inside the cells are a mixture of tri-phosphorylated (primary) transcripts, and mono-phosphorylated (processed) transcripts. The 5'-bases of the mono-phosphorylated RNAs are marked with asterisks, and the downstream fragment generated by an RNase Y cleavage is in red (absent from the ΔY strain). The two total RNA samples, one from the WT strain and one from the ΔY mutant are separately ligated to the Rp6 oligo (light blue), a reaction that only works for 5' mono-phosphorylated RNA. The Rp6 RNA oligo has 7 random bases, the so-called Quantification Sequence, in the middle (indicated by NN). The ligated RNA is reverse transcribed with a primer which has a random sequence near the 3' end, and a tag (light green) at the 5' end. This generates cDNA with a known sequence at the 5' end. At the 3' end, a cDNA molecule will also have a known sequence, if (and only if) the reversely transcribed RNA molecule was ligated to the Rp6 oligo (i.e. the RNA molecule was mono-phosphorylated in the cell). The cDNA that has the cRp6 tag at one end and the reverse transcription tag at the other end is then PCR-amplified with primers that add the appropriate adaptor-sequences for Illumina sequencing at the ends (Illumina 1.0 and Illumina 2.0, marked in dark blue and dark green respectively). Additionally, the primers that hybridises to the cRp6 sequence contain an EMOTE barcode (A and B) that is unique for each RNA sample. Once the EMOTE barcode has been added, then the PCR products can be mixed, since each and every molecule can be bioinformatically traced back to the original RNA sample it came from. The mix of PCR-products is then sequenced, using Illumina technology, from the “Rp6-end”. As little as 50 nt sequence will read through the EMOTE barcode and Quantification Sequence, into the first 20 nt of the original mono-phosphorylated RNA molecule. These 20 nt of transcriptome sequence, referred to as the Mapping Sequence, can be aligned to the place on the genome that corresponds [file pgen.1005577.s003.pdf]

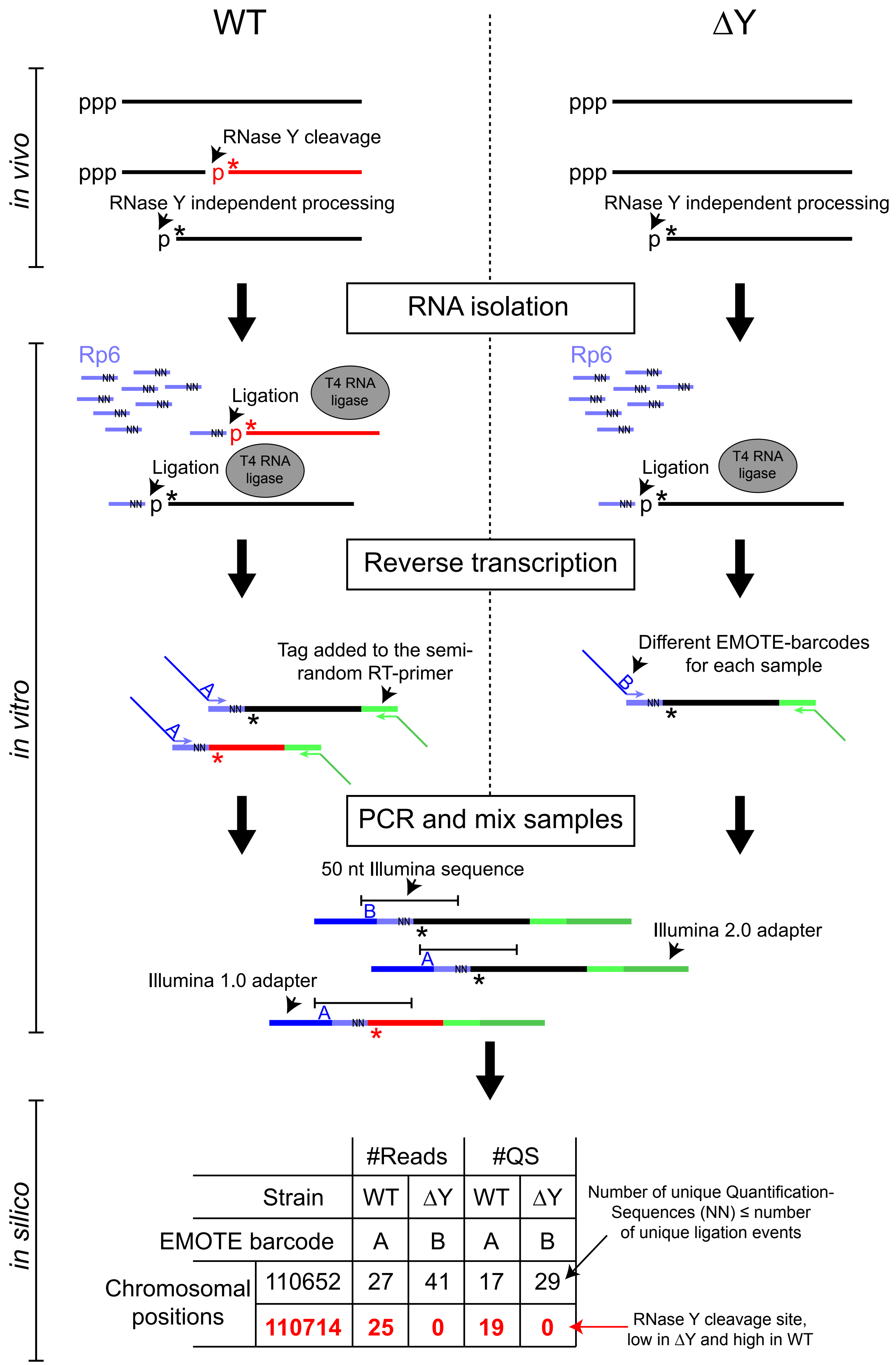

WT
ΔY
in vivo
ppp
RNase Y cleavage
p
RNase Y independent processing
RNA isolation
in vitro
Rp6
NN
Ligation
T4 RNA ligase
Reverse transcription
Tag added to the semi-random RT-primer
Different EMOTE-barcodes for each sample
A
B
PCR and mix samples
50 nt Illumina sequence
Illumina 2.0 adapter
Illumina 1.0 adapter
in silico
#Reads
#QS
Strain
WT
ΔY
WT
ΔY
EMOTE barcode
A
B
A
B
Chromosomal positions
110652
27
41
17
29
110714
25
0
19
0
Number of unique Quantification-Sequences (NN) ≤ number of unique ligation events
RNase Y cleavage site, low in ΔY and high in WT
